# Supplementary material for: Direct Observation of the Interconversion of Normal and Toxic Forms of α-Synuclein
Source: Cell. 2012 May 25;149(5):1048–59. doi: 10.1016/j.cell.2012.03.037 (PMC3383996; doi:10.1016/j.cell.2012.03.037)
Supplement: Table S1. Relative Percentage of the Different Oligomeric Classes, Related to Figure 4 [file mmc1.pdf]

Table S1. Relative Percentage of the Different Oligomeric Classes, Related to Figure 4

Oligomer class:	A_{small}	A_{med}	B_{med}	B_{large}
Late aggregation time	28 ± 4	20 ± 1	27 ± 1	25 ± 2
Early disaggregation time	48 ± 12	9 ± 8	31 ± 15	11 ± 2
