# Supplementary material for: Direct Observation of the Interconversion of Normal and Toxic Forms of α-Synuclein
Source: Cell. 2012 May 25;149(5):1048–59. doi: 10.1016/j.cell.2012.03.037 (PMC3383996; doi:10.1016/j.cell.2012.03.037)
Supplement: Document S1. Article plus Supplemental Information [file mmc2.pdf]

Cell

Direct Observation of the Interconversion of Normal and Toxic Forms of α-Synuclein

Nunilo Cremades,[1] Samuel I.A. Cohen,[1] Emma Deas,[2] Andrey Y. Abramov,[2] Allen Y. Chen,[1] Angel Orte,[1,3] Massimo Sandal,[1] Richard W. Clarke,[1] Paul Dunne,[1] Francesco A. Aprile,[1,4] Carlos W. Bertoncini,[1,5] Nicholas W. Wood,[2] Tuomas P.J. Knowles,[1] Christopher M. Dobson,[1,*] and David Klenerman[1,*]
[1]Department of Chemistry, University of Cambridge, Lensfield Road, Cambridge CB2 1EW, UK
[2]Department of Molecular Neuroscience, University College London, Institute of Neurology, Queen Square, London WC1N 3BG, UK
[3]Department of Physical Chemistry, Faculty of Pharmacy, University of Granada, Campus Cartuja, 18071 Granada, Spain
[4]Department of Biotechnology and Biosciences, University of Milano-Bicocca, Piazza della Scienza 2, 20126 Milan, Italy
[5]Institute for Research in Biomedicine, Baldiri Reixac 10, 08028 Barcelona, Spain
*Correspondence: cmd44@cam.ac.uk (C.M.D.), dk10012@cam.ac.uk (D.K.)
DOI 10.1016/j.cell.2012.03.037

SUMMARY

Here, we use single-molecule techniques to study the aggregation of α-synuclein, the protein whose misfolding and deposition is associated with Parkinson's disease. We identify a conformational change from the initially formed oligomers to stable, more compact proteinase-K-resistant oligomers as the key step that leads ultimately to fibril formation. The oligomers formed as a result of the structural conversion generate much higher levels of oxidative stress in rat primary neurons than do the oligomers formed initially, showing that they are more damaging to cells. The structural conversion is remarkably slow, indicating a high kinetic barrier for the conversion and suggesting that there is a significant period of time for the cellular protective machinery to operate and potentially for therapeutic intervention, prior to the onset of cellular damage. In the absence of added soluble protein, the assembly process is reversed and fibrils disaggregate to form stable oligomers, hence acting as a source of cytotoxic species.

INTRODUCTION

α-synuclein (αS) is a 140 residue protein expressed abundantly in the brain, where it can account for up to 1% of all proteins in the neuronal cytosol (Bonini and Giasson, 2005). It does not appear to be an essential protein (Abeliovich et al., 2000) and has multiple proposed functions interacting specifically with numerous proteins involved in signal transduction, vesicular trafficking, synaptic behavior, the regulation of oxidative stress, and mitochondrial function (Bonini and Giasson, 2005). It is, however, the major constituent of intracellular protein-rich inclusions, Lewy bodies and Lewy neurites, the hallmark lesions of Parkinson's disease (PD) (Spillantini et al., 1998). These inclusions share common structural characteristics, including a high β sheet content and a distinctive cross-β X-ray diffraction pattern, also observed for fibrillar deposits associated with other degenerative disorders, including Alzheimer's disease, and commonly described as amyloid fibrils (Geddes et al., 1968; Chiti and Dobson, 2006).

The formation of amyloid fibrils in vitro is a common phenomenon and is usually monitored through measurements of turbidity or by means of fluorometric dyes, such as Thioflavin T. These experiments suggest that fibril formation follows a nucleation-polymerization model (Jarrett and Lansbury, 1992), where soluble species undergo a nucleation process that generates oligomeric species that are then able to grow through further monomer addition, thereby forming protofilaments and eventually mature fibrils. The characteristic sigmoidal growth profile reflects the greater ease of addition of monomers onto existing aggregates compared with the de novo formation of new oligomers directly from monomers alone. The overall reaction rate therefore accelerates when significant numbers of aggregates are present in solution, resulting in an initial lag phase followed by a growth phase during which the overall conversion is accelerated before a plateau region is reached when the monomer concentration is depleted (Figure 1A). Recently, however, it has become evident that the kinetics of fibril growth can often be dominated by secondary nucleation events, such as fibril fragmentation (Cohen et al., 2011; Knowles et al., 2009), adding further elements of complexity to the kinetic process.

Both experimental and theoretical studies of the kinetics of fibril formation have given important insights into the overall mechanism of amyloid assembly, but little is known in any detail about the oligomeric species that not only represent the crucial first steps of the self-association process but have also been implicated as key species in the pathogenesis of protein misfolding and deposition diseases (Bucciantini et al., 2002; Kayed et al., 2003; Lashuel et al., 2002; Luheshi et al., 2007; Tokuda et al., 2010; Winner et al., 2011). A variety of distinct morphologies of αS oligomers have been observed using

 ©2012 Elsevier Inc.

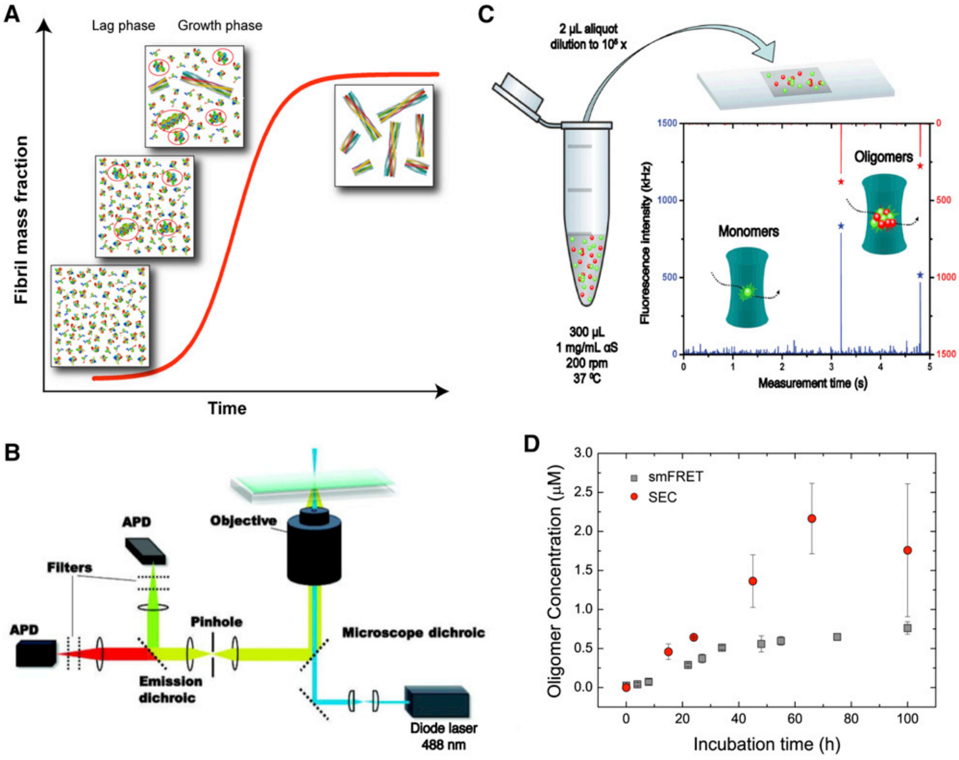

Figure 1. Experimental Protocol

(A) Example of the kinetics of amyloid fibril formation, including hypothetical snapshots of the ensemble of αS species present at different phases of the aggregation process, wherein the oligomeric species present in the sample are highlighted with red circles.

(B) Schematic representation of the instrument used for smFRET measurements.

(C) Schematic description of the experimental protocol for aggregation experiments. Bursts of fluorescence coincident in both channels indicate the presence of FRET-positive oligomeric species (marked as asterisks). Noncoincident bursts can be attributed to monomers and are normally much less bright than those corresponding to oligomers.

(D) Comparison between the kinetics of oligomer formation under bulk conditions obtained by quantitative SEC analysis (red circles; see also Figure S1G) and from smFRET experiments, after extrapolating the concentrations from sm to bulk conditions (gray squares). The data reported correspond to the mean and standard errors of two repetitions in the case of the SEC data and five repetitions for the sm data. See Figure S1 for a detailed characterization of the effect of the fluorophores on αS aggregation.

imaging techniques, notably, atomic force microscopy or transmission electron microscopy (see, e.g., Conway et al., 2000; Ding et al., 2002; Lashuel et al., 2002; Hoyer et al., 2004). Structural studies on αS oligomers have also been carried out using FTIR, Raman, CD, and fluorescence spectroscopy (see, e.g., Apetri et al., 2006; Goldberg and Lansbury, 2000; Hong et al., 2008; Nath et al., 2010; Thirunavukkuarasu et al., 2008), which have revealed the formation of various oligomeric structures during αS aggregation, consistent with a progressive increase in β sheet structure occurring concomitantly with the formation of more ordered aggregates.

In order to define in more detail the types of oligomers formed during αS fibril formation and the rates at which they develop, it is necessary to find the means of overcoming the challenges inherent in studying these heterogeneous and frequently transient intermediate species. Recently, techniques have been developed that are able to observe individual molecular species in solution (Chiou et al., 2009; Kostka et al., 2008; Orte et al., 2008), and in the present work we describe experimental and theoretical approaches that enable us to define in microscopic detail the assembly of αS into amyloid fibrils. As we discuss below, the results obtained shed new light on the underlying molecular processes involved in αS oligomerization and fibril formation and, in conjunction with cell biology experiments, provide crucial information on their link with pathogenesis.

RESULTS AND DISCUSSION

Single-Molecule Experiments Reveal the Presence of Distinct Oligomeric Species during αS Fibril Formation

We have previously demonstrated, using a model amyloidogenic protein, that it is possible to quantify the fraction of oligomers in a sample and estimate their size distribution using the two-color coincidence detection (TCCD) method in diffusion single-molecule (sm) fluorescence experiments (Orte et al., 2008). Here, we have applied the same principles to αS and have selected residue 90, at the C-terminal end of the central hydrophobic NAC region of αS, as an appropriate position to incorporate fluorophores (see Figure S1 available online). Although this residue has previously been tagged with

fluorescent probes to investigate the aggregation of αS (Thirunavukkuarasu et al., 2008), we nevertheless compared the aggregation behavior of αS tagged with the two fluorophores used in the TCCD experiments, Alexa Fluor 488 (AF488) and Alexa Fluor 647 (AF647), to that of the unlabeled protein and established that the presence of the dye molecules does not alter the fundamental features of the aggregation reaction or the appearance of the resulting fibrillar aggregates (see Extended Experimental Procedures for details).

We next performed a series of smTCCD experiments at different time points during the αS aggregation process. Equimolecular mixtures of αS labeled with either AF488 or AF647 were incubated under conditions used previously to form fibrils in vitro (Wood et al., 1999), and aliquots were taken at a series of time points and then diluted rapidly by a factor of 10^5 to a final concentration of ~500 pM, which is suitable for sm analysis. In TCCD, the confocal volume is excited by the overlapped red (633 nm) and blue (488 nm) lasers, and oligomers are detected as coincident bursts of fluorescence and readily distinguished from single-label monomers once account is taken of chance coincidental background events (Orte et al., 2006). In this way, we were able to estimate the fraction of oligomeric species along the αS aggregation process, as well as track the time evolution of their size distribution.

To obtain additional information on the nature of the oligomers formed in the aggregation process, we explored the use of intermolecular fluorescence resonance energy transfer (FRET) measurements. The samples were irradiated by the blue laser alone (Figure 1B), that is, only AF488-labeled molecules were excited directly. We observed, however, that the AF647 dye was excited indirectly by FRET from adjacent AF488 fluorophores within the aggregated species, generating coincident fluorescence events in both the AF488 and AF647 emission channels (Figures 1C and S2A); monomers, by contrast, can only give rise to bursts of fluorescence in the AF488 emission channel. This approach therefore allowed us to quantify the relative number of oligomeric and monomeric species in each sample and explore if there are any differences in the FRET efficiency that would indicate differences in the oligomer structure.

This analysis was then applied to samples taken at different incubation times during the aggregation reaction, enabling us to characterize the populations of αS oligomeric species as a function of their FRET efficiency and size. Because size is determined indirectly from fluorescence measurements in confocal single-molecule techniques applied in solution, we refer to the apparent oligomer size throughout the paper, which is only an estimate of the real oligomer size, in part due to the different paths that an oligomer can take through the confocal volume, but it can be used to determine the differences in relative sizes between oligomeric species. Based on our previous method (Orte et al., 2008), we determined the apparent size of αS oligomers by comparing the intensity in the AF488 emission channel of the fluorescence burst due to a given oligomer with the average intensity value for the AF488-labeled monomer (fluorescence lifetime measurements confirm that there is no detectable fluorophore quenching in monomers or soluble oligomers; see Extended Experimental Procedures) after correcting for the average FRET efficiency of that particular oligomer (see Experimental Procedures). Both the fraction of oligomers and their size distribution during aggregation were in very good agreement with those obtained by smTCCD measurements.

A representative set of smFRET results is shown in Figure 2A. The data have been analyzed to generate two-dimensional (2D) histograms, in which the distribution of apparent oligomer sizes is represented as a function of FRET efficiency. Incubation of monomeric αS under the aggregation-promoting conditions used here initially results in the formation of a series of small oligomers ($n \leq 10$) and then, after a period of a few hours, in a wide distribution of oligomers, ranging in size from dimers to species containing more than 100 monomers. Despite the highly heterogeneous size distribution of the oligomeric species, a relatively small range of FRET efficiency values is observed. From a visual inspection of the data (Figure 2B, top), the distribution of FRET values appears to vary with the size of the oligomers, revealing two dominant populations, one corresponding to those of small size with medium FRET values (0.4–0.7) and the other to larger species with higher FRET values (0.6–0.9). To analyze these populations in more detail, we classified the oligomers into three broad size groups, small (~2–5 mers), medium (~5–15 mers), and large (~15–150 mers) (larger oligomers were excluded from the analysis, because too few were encountered to generate significant statistics), and fitted the FRET histograms for each group as Gaussian distributions (see Extended Experimental Procedures for a detailed description of the method).

Using this classification we detected four distinct distributions of oligomers, denoted as A_{small}, A_{med}, B_{med}, and B_{large}, where A and B are used to distinguish between species with mid- and high-FRET values, respectively, and the subscripts are used to distinguish between the different oligomer size groups. Further examination of the data revealed that the mean and the width of the FRET distributions for each oligomer group remained unchanged during the incubation time, allowing the data sets at the different times to be fitted globally (Figure 2C). To test the robustness of this approach, five repetitions of the entire aggregation experiment were carried out, and the same four populations with almost identical Gaussian parameters defining their FRET distributions were obtained in each case (see Table 1 and error bars in Figure 3A).

We also analyzed the formation of oligomers at a protein concentration (6 μM) similar to that found under physiological conditions (Iwai et al., 1995). Interestingly, the two major structural types of oligomers detected at a concentration of 70 μM were also found in the experiments carried out at this lower concentration (Figure S2B), although their absolute levels were much lower and no fibrils were detectable by transmission electron microscopy even after 7 days of incubation. Crucially, this finding suggests that at physiologically relevant concentrations (Iwai et al., 1995), αS is able to form potentially toxic oligomeric species.

Next, we set up a series of smFRET experiments on samples diluted in the presence of unlabeled αS and compared the number and type of oligomers detected with those found in samples diluted into buffer alone. The relative difference in the number of oligomers detected in the two cases provides an estimate of the relative kinetic stabilities of the oligomeric species upon dilution to single-molecule conditions. We

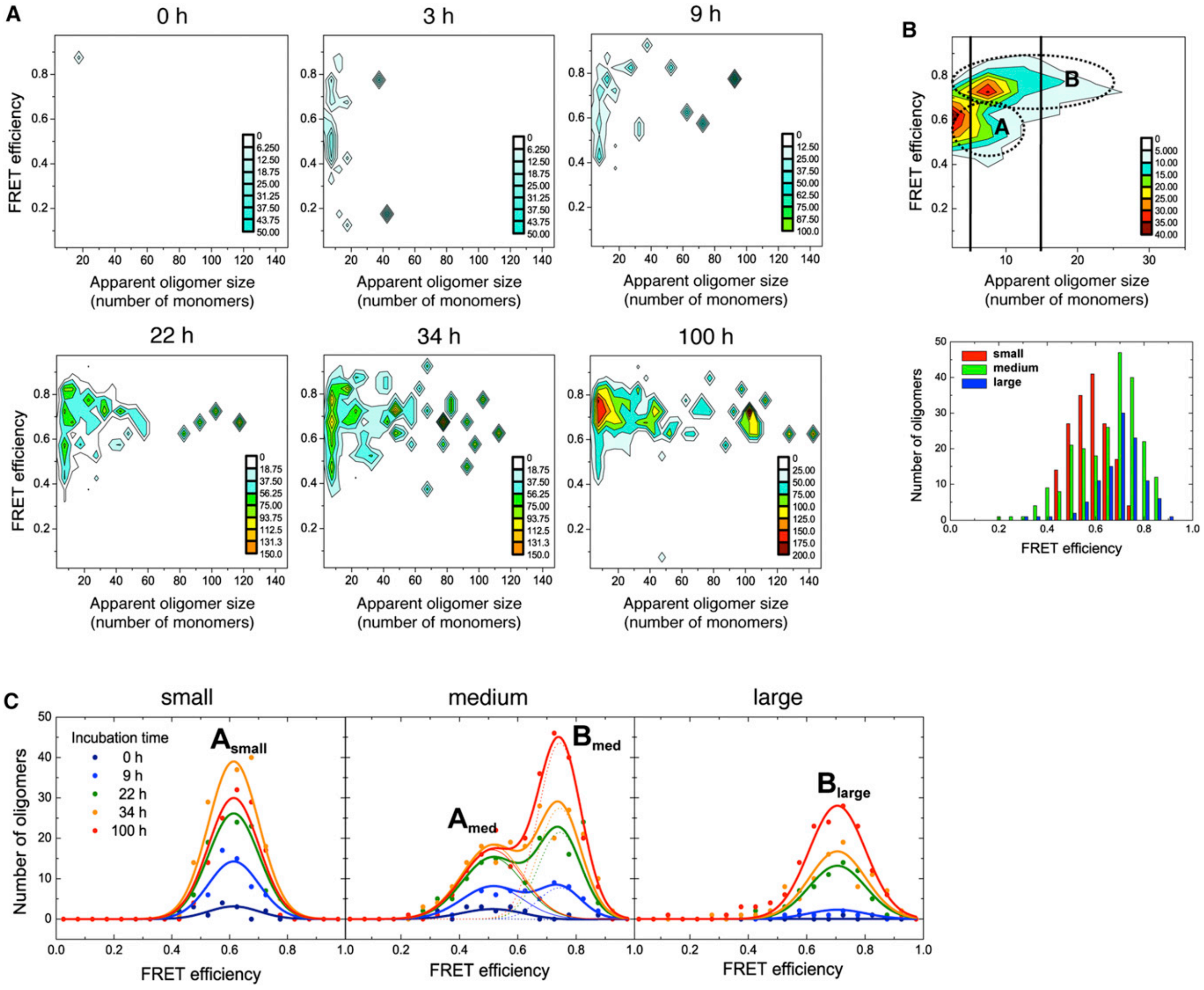

Figure 2. αS Oligomers Present during Fibril Formation

Aggregation of αS was followed by smFRET at a range of time points during incubation.

(A) 2D plots corresponding to the relative mass distribution of oligomers at different incubation times as a function of apparent oligomer size (x axis) and FRET efficiency (y axis); for a clearer visualization of the presence of large oligomers, the mass rather than the number distribution is represented.

(B) A representative 2D plot of the number distribution of oligomers after 60 hr of incubation illustrates the size-dependence of the FRET efficiency distributions (vertical lines corresponding to apparent oligomer sizes of 5- and 15-mer have been added as a visual guide), where two main FRET oligomer populations can be identified. The FRET-derived distributions based on small (red bars), medium (green bars), and large (blue bars) classification are plotted on the graph shown below.

(C) Representative global fits of the four size-derived FRET oligomer distributions to Gaussian functions as the incubation time varies. See also Figure S2.

detected the same FRET distributions and comparable numbers of oligomers in both types of experiments (data not shown), far from the difference of a factor of 10^5 expected from the dilution from bulk to single-molecule conditions if the oligomers were to dissociate rapidly at pM concentrations.

This assumption was further strengthened by the close agreement between the concentrations of oligomers detected under bulk conditions by quantitative size-exclusion chromatography (SEC) and those estimated from our single-molecule experiments and extrapolated to bulk conditions at different incubation times (Figure 1D). In the SEC analysis under bulk conditions, we were able to quantify directly the concentrations of oligomers present at different incubation times by exploiting the high extinction coefficients of the fluorophores in the visible region of the spectra (Figure S1G). The mass fraction of soluble aggregates was found at all times to be very low (≤5%), in agreement with a previous report (Conway et al., 2000), and the kinetics of oligomer formation were in good agreement with extrapolations from the single-molecule experiments (Figure 1D). The discrepancies at long incubation times between the two set of data can be attributed to the increasing contribution to the mass fraction of large oligomers formed at long incubation times,

Table 1. Kinetic Analysis of the Formation of Different Oligomeric Populations Classified by Apparent Size and FRET Efficiency during αS Aggregation and Fibril Formation

Oligomer Type	Apparent Growth Rate (hr^{-1})	Lag Time (hr)	Average FRET Efficiency
A_{small}	0.047 ± 0.019	0.2 ± 1.7	0.61 ± 0.02
A_{med}	0.041 ± 0.013	0.1 ± 1.0	0.53 ± 0.05
B_{med}	0.013 ± 0.005	2.7 ± 1.2	0.74 ± 0.03
B_{large}	0.013 ± 0.003	3.3 ± 1.4	0.68 ± 0.05

The apparent growth rate and the lag time were obtained from the quantification of each oligomer type obtained by smFRET experiments by fitting the time dependence of the population of each oligomer class to the function $y = A \cdot (1 - \exp(-(x - x_0) \cdot r))$, where A is the amplitude, r is the apparent growth rate, and x_0 is the lag time. The data and the fits to this function are plotted in Figure 3A (see Extended Experimental Procedures for a detailed explanation of the analysis). The errors reported for the kinetic parameters correspond to the fitting errors. The average FRET efficiency was obtained from a global analysis of the FRET distributions at the different incubation times (see Figure 2C). The values reported correspond to the mean and standard error of five repetitions.

which were excluded from the analysis in our smFRET experiments because of inefficient sampling of these species.

αS Oligomers Undergo Structural Reorganization prior to Fibril Growth

We next determined the kinetics of formation of all four types of oligomers observed in the smFRET experiments (Figure 2C). For simplicity, we fitted a single-exponential function coupled with a lag time (Figure 3A); the parameters obtained from the fitting are shown in Table 1. Oligomers A_{small} and A_{med} show similar kinetics, as do oligomers B_{med} and B_{large} (see Table 1), suggesting that the different FRET efficiencies report on two different structural types of oligomers. A longer lag time was observed for the formation of type B oligomers, suggesting that these species could result from a reorganization of type A oligomers, in agreement with previous suggestions that fibril formation by αS involves the sequential appearance of discrete oligomeric intermediates (Conway et al., 2000).

To gain further insight into the different structures of A and B oligomers, we measured the susceptibility of each to proteinase K degradation relative to that of the monomeric and fibrillar forms of the protein. The core of αS fibrils, like that of other amyloid structures, is highly resistant to proteinase K degradation (Miake et al., 2002; Neumann et al., 2002). We found that the fibrils labeled with fluorophores were completely resistant to degradation when treated with 0.4 μg/ml proteinase K for 20 min at 37°C but were gradually degraded when the proteinase K concentration was increased from 0.4 to 10 μg/ml (Figure S3B), in agreement with the proteinase K degradation profiles reported previously for αS fibrils both in vitro and in vivo (Neumann et al., 2002). In contrast, the monomeric form of the protein is highly susceptible to degradation in agreement with expectations in the light of its intrinsically disordered nature.

The resistance to degradation of A- and B-type oligomers was assessed by smFRET experiments after similar incubations at increasing proteinase K concentrations (see Extended Experimental Procedures and Figure S3A). By comparing the concentrations of proteinase K at which 50% of each protein species is degraded (Cm), we can conclude (Figure 3B) that type-A oligomers are as sensitive to proteinase K as the monomeric protein (Cm = 0.05 ± 0.09 μg/ml, and 0.05 ± 0.01 μg/ml, respectively), whereas type-B oligomers are substantially more resistant to degradation (Cm = 0.27 ± 0.02 μg/ml) although not as highly resistant as the mature amyloid fibril (Cm = 1.1 ± 0.3 μg/ml). As resistance to proteolytic, and specifically proteinase K, digestion has been found generally to correlate with the degree of persistent structure and β sheet content of aggregated species (Kocisko et al., 1995; Miake et al., 2002; Nordstedt et al., 1994), our results strongly suggest that type-A oligomers lack persistent structure, although their structure cannot be random, because they give rise to a distinct FRET peak. In contrast, type-B oligomers are likely to contain a significant degree of β sheet structure. This conclusion is in agreement with previous studies of αS aggregation, where a progressive increase in β sheet structure was observed during αS aggregation before the formation of mature amyloid fibrils (Apetri et al., 2006).

Disaggregation of αS Fibrils Leads to the Release of Oligomeric Species

A number of studies have shown directly or indirectly that amyloid fibrils are able to disaggregate, for example, as a result of changes in pH (Picotti et al., 2007) or addition of chemical denaturants (Calamai et al., 2005; MacPhee and Dobson, 2000). We therefore set out to probe the disaggregation of αS fibrils under near-physiological conditions by incubating preformed labeled αS fibrils at 37°C in monomer-free buffer (see Experimental Procedures) and carrying out a series of smFRET experiments over a period of several weeks (Figure 4). There was greater variability in the data from these experiments than from the aggregation experiments, an observation attributable to a significant variability in the degree of maturation of the fibrils in the different samples. We observed in all cases that, however, disaggregation results in species with a wide variation in size (Figure 4), indicating that monomeric, as well as oligomeric, species detach from the fibrils; after longer incubation times, however, these oligomers were found to have dissociated into monomers (see Figures 4, S4A, and S4B). Interestingly, a high fraction of oligomers formed at the beginning of the disaggregation experiment possesses FRET efficiencies similar to the type-B oligomers that are formed in the late stages of aggregation (see Table S1).

Because standard confocal smFRET techniques applied in solution have limitations in the detection of large species, we used single-molecule total internal reflection fluorescence (smTIRF) experiments to probe further the nature of the aggregates generated by fibril disaggregation and in the late stages of the aggregation process (see Extended Experimental Procedures and Figures S4C–S4F). Under the latter conditions, two FRET distributions were observed, corresponding to the two distributions detected in solution by smFRET experiments (type A and B), whereas in the fibril disaggregation sample, at early incubation times, the data show essentially a single distribution at high-FRET efficiencies, corresponding to type-B oligomers. Thus, the TIRF experiments confirm that fibrils initially disaggregate largely into type-B oligomers, a finding

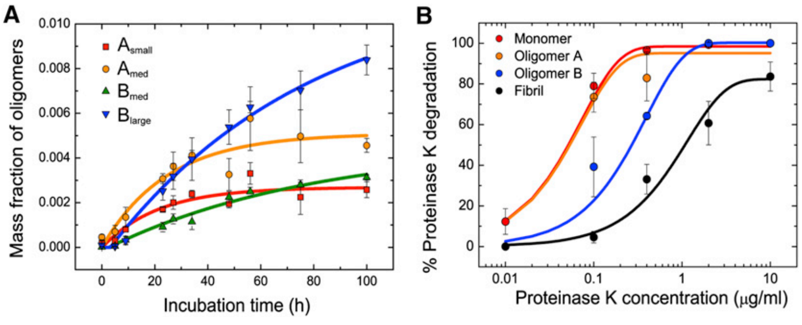

Figure 3. Characterization of the Different Oligomeric Species Formed during αS Aggregation and Fibril Formation

(A) The time dependence of the mass fraction of the four oligomeric distributions A_{small} (red squares), A_{medium} (orange circles), B_{medium} (green triangles), and B_{large} (blue triangles). The data shown correspond to the average and standard error of five different experiments.

(B) Proteinase K degradation curves of the different protein species (monomer in red, type A oligomer in orange, type B oligomer in blue, and fibrils in black). The data shown here correspond to the average and standard error of three different experiments. See also Figure S3.

that strongly supports the view that the latter possess a β sheet structure that could resemble that found in the amyloid state. Moreover, the fact that both types of oligomers are detected in fibril disaggregation experiments provides further evidence for a structural conversion between the two types of oligomers and that such a conversion is likely to be a key step in fibril formation.

Kinetic Analysis of the Early Stages of Amyloid Fibril Formation

We then set out to determine the rate constants for the reactions associated with the early stages of aggregation using our smFRET experiments. For this purpose, we needed a kinetic model that could explain the following experimental observations: (1) a lag phase in the formation of fibrils; (2) the presence of two different structural types of oligomers; and (3) a lag phase in the appearance of type-B, but not type-A, oligomers.

A general nucleation and growth model can explain the lag phase observed in bulk αS fibril formation experiments. This model then needs to include a conformational conversion step to take account of the two types of oligomeric states observed during aggregation and of the lag phase for type-B oligomers. The simplest model that is consistent with these observations is a nucleation and growth model with a conformational conversion step, analogous to that used to describe the conformational conversion mechanism suggested for prions (Serio et al., 2000). We therefore extended the analytical solution that we have generated to interpret the kinetic rate equations for a nucleation and growth model (Knowles et al., 2009) by the addition of a conversion step. As shown in Figure 5A, this model assumes that primary nucleation results in the creation of oligomers of type A from monomeric protein molecules. Type-A oligomers can grow through monomer addition, but they can also convert into type-B oligomers, indicating that multiple parallel pathways could result in the formation of type-B oligomers of a given size; details of the early-time analytical and exact numerical solutions of the model are presented in the Supplemental Information.

This model was then used to fit the experimental kinetic data describing the formation of the two types of oligomers over the first 30 hr of incubation, that is, during the lag phase observed in the kinetics of formation of fibrils under bulk conditions; as shown in Figure 5B, the model fits the data well. At later times, additional events, such as the formation of large fibrillar species, the contribution of the reverse processes involving conversion of type-B oligomers into type-A oligomers, and the dissociation of type-A oligomers, become significant and result in underfitting (see Extended Experimental Procedures). Overall, therefore, this kinetic description in terms of the dominant events associated with both fibril formation and disaggregation enables the data obtained from single-molecule experiments to be understood on the basis of a small number of well-defined microscopic parameters, the values of which can be determined directly from the experimental data. Thus, the primary nucleation rate constant for oligomers of type A can be defined as $\sim 4 \times 10^{-8}\ s^{-1}$ and the conversion rate constant from type-A to type-B oligomers as $\sim 5 \times 10^{-6}\ s^{-1}$. Note that, because the number of monomeric species able to participate in primary nucleation greatly exceeds the number of oligomers that are involved in the structural conversion, the overall primary nucleation rate is larger than the conversion rate, a situation that results in the observed build-up of the type-A oligomers during the aggregation process.

A particularly interesting result is that the rate of structural conversion between the two types of αS oligomers determined from the analysis corresponds to a half-time of ca. 35 hr, a time which is very long compared, for example, with the time required for the folding of small single-domain globular proteins, the characteristic times for which range from microseconds to seconds (Jackson, 1998). The slow rate indicates that the energy barrier for the conversion from a relatively unstructured oligomer to a β sheet rich oligomer is very high, an observation that can be attributed in part to the multiple permutations of interstrand hydrogen bonding interactions and in part to the fact that the β sheet rich species are likely to have a significant fraction of their hydrophobic side chains exposed to the solvent as found in amyloid fibrils (Heise et al., 2005; Vilar et al., 2008). Furthermore, previous studies of the folding kinetics of pertactin, a protein that has an elongated parallel β-helix shape resembling that of an amyloid fibril, found a half-time for refolding of 3 hr (Junker and Clark, 2010), only an order of magnitude faster than the rate of reorganization of oligomeric αS into intermolecular β sheet structures, suggesting that the intrinsic formation of an amyloid-like structure proceeds through an energy landscape with significant barriers between different regions of conformational space, in contrast to the smooth funnel-like landscape found for the majority of small naturally evolved proteins (Onuchic et al., 1997; Dinner et al., 2000).

PK-Resistant Oligomers Induce Higher Aberrant Levels of ROS in Cells Than Do PK-Sensitive Oligomers

Given the long-standing pathophysiological link between increased levels of oxidative stress and PD (Jenner and Olanow,

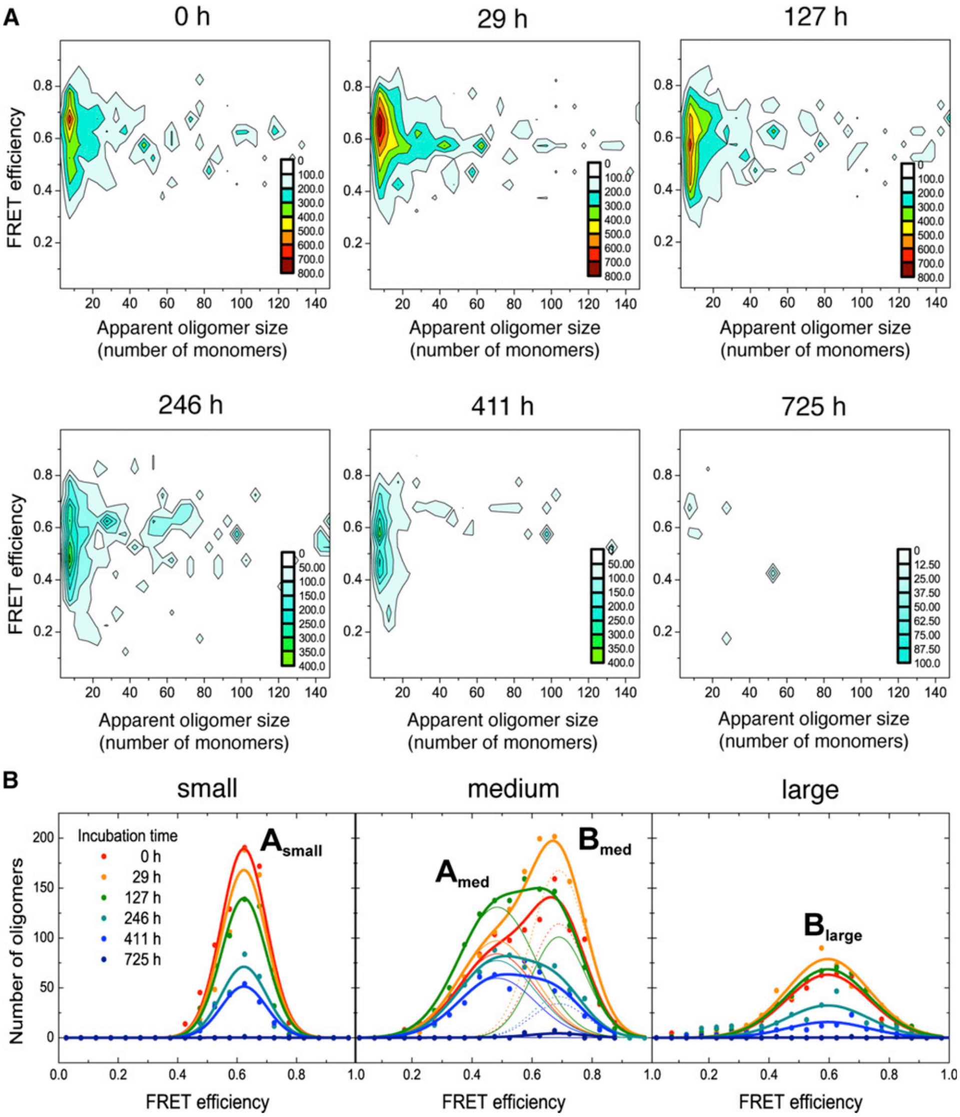

Figure 4. αS Fibril Disaggregation

(A) 2D plots of the relative mass distribution of oligomers according to apparent size and FRET efficiency, as explained in Figure 2A.

(B) Representative global fits of the size-derived FRET oligomer distributions as a function of the incubation time. See also Figure S4 and Table S1.

1998; Jenner, 2003) and the reported specific generation of intracellular reactive oxygen species (ROS) by aggregating proteins implicated in neurodegenerative diseases (Tabner et al., 2001; Parihar et al., 2009), we examined if the production of ROS changed on exposure of rat primary midbrain neurons to samples containing different αS species, using samples from identical aggregation experiments to those described above. Two samples containing a mixture of monomeric and oligomeric species with different concentrations of type-A and type-B oligomers were obtained by taking aliquots of an aggregating sample at two different incubation times. The first sample was enriched in type-A oligomers (denoted as the "oligomer A sample") and was obtained after 20–25 hr of incubation; it contained ca. 200 nM of type-A oligomers and ca. 50 nM of type-B oligomers (concentrations are in number of species) in the presence of 70 μM of monomers. The second sample (denoted the "oligomer B sample"), taken after 70 hr of incubation, contained ca. 200 nM of type-A oligomers and ca. 150 nM of type-B oligomers in the presence of 50 μM of monomers. In both cases, fibrillar species were removed from the sample by centrifugation. In addition, we took an aliquot from the sample before initiating the aggregation as a monomeric control sample (at a concentration of 70 μM) and another aliquot at the end of the aggregation as a fibrillar control sample. The latter was centrifuged and the fibrillar material resuspended in the same volume as that of the initial sample, which contained ca. 200 nM of fibrils (in number concentration).

We first assessed the ability of cells to take up the different forms of αS using confocal microscopy; the rate of uptake of the differently labeled αS species was calculated by measuring

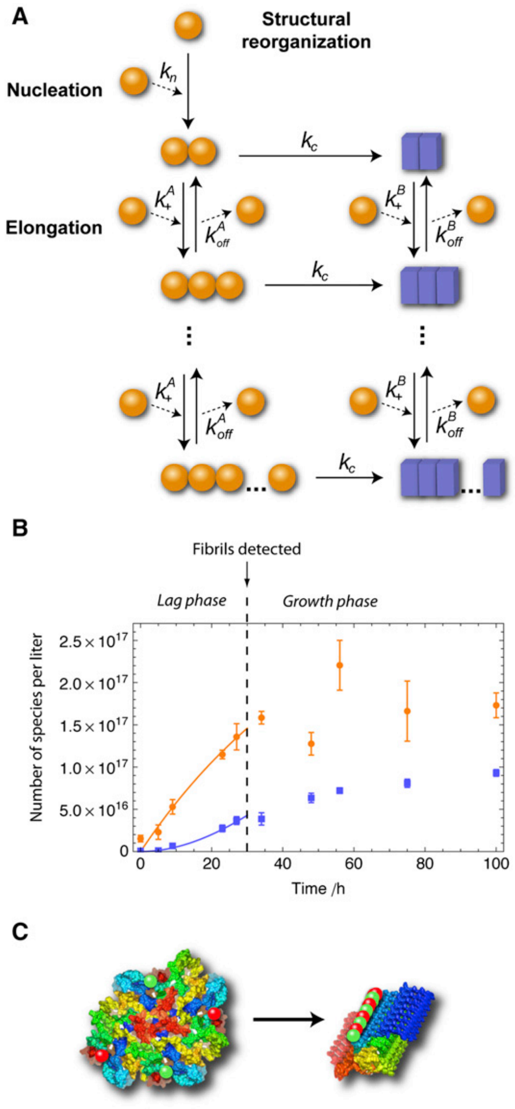

Figure 5. Kinetic Analysis of αS Oligomerization

(A) Scheme for the minimalistic kinetic model used to fit the early stages of αS aggregation.

(B) Results of the global fitting (continuous lines) of the kinetics of formation of the two types of oligomeric species estimated under bulk conditions from smFRET experiments. Data for type A oligomers and type B oligomers are shown as orange circles and blue squares, respectively (average and standard error of five different experiments). The vertical dashed line is at 30 hr, corresponding to the lag phase of fibril formation estimated under bulk conditions, up to which time our model accounts well for the different microscopic processes governing the aggregation reaction.

(C) Cartoon showing the conversion of an 8-mer of αS from a collapsed to an ordered proteinase-K-resistant form. Residues of each monomer are colored according to their location in the amino acid sequence. The average distance between fluorophores, represented as green and red spheres, is different for each type of oligomer and hence gives rise to different average FRET efficiencies.

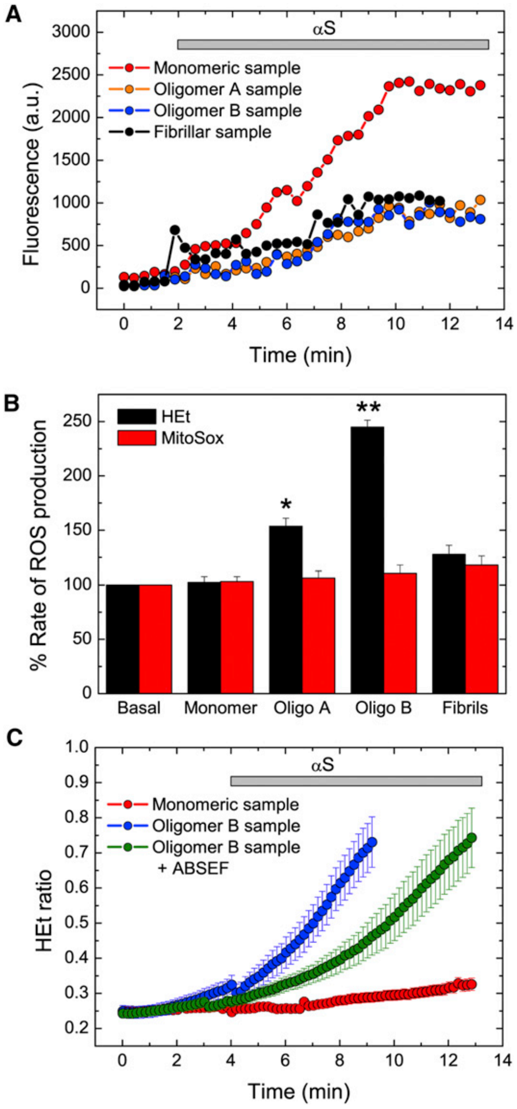

Figure 6. Evaluation of the Pathophysiological Effects of the αS Species on Rat Primary Neuronal Cultures

(A) Representative traces showing the rate of uptake of the different types of αS species into the cell body. The fluorescence of AF488-labeled αS was used to track monomeric αS uptake, whereas the fluorescence emission of AF647-labeled αS was used to follow the uptake of oligomeric and fibrillar forms of the protein (see also Figure S5), as only these species are able to produce FRET signals.

(B) Bar chart displaying cytoplasmic (HEt) and mitochondrial (MitoSOX) ROS production induced in cells after exposure to each αS sample (see "PK-Resistant Oligomers Induce Higher Aberrant Levels of ROS in Cells than Do PK-Sensitive Oligomers" section in the main text for a detailed explanation of the composition of each sample). $^*p < 0.05$; $^{**}p < 0.005$.

(C) Representative cellular traces showing the slower production of ROS induced by type B αS oligomeric samples after cellular treatment with ABSEF. Error bars in (B) and (C) represent SEM.

the increase in the fluorescent intensity signal within the cell body (Figures 6A and S5). We observed a rapid (between 6–8 min) and efficient uptake of all αS species into both neurons and astrocytes. The exposure of the cells to the αS species had no detectable effect on cell viability over 24 hr. For the monomeric protein, the time for maximal uptake reported here

corresponds to the time to reach saturation in the quantity of internalized protein, whereas for oligomeric and fibrillar species, due to the reduced quantity of these species in the corresponding solutions, it represents the time the cells needed to take up the total number of FRET-positive species added to the cellular media (see Figure S5).

For cytosolic ROS assessment, cultures were briefly incubated in the presence of hydroethidium (HEt) prior to exposure to αS samples, whereas cultures were preincubated with MitoSOX (the mitochondrially targeted equivalent of HEt) for mitochondrial ROS measurements. Exposure of the cultures to 10 μl monomeric or fibrillar αS samples had a marginal and insignificant effect on cytoplasmic and mitochondrial ROS production (Figure 6B). By contrast, exposure of the neuronal cultures to the type-B oligomeric samples, and to a lesser degree to the type-A oligomeric samples, induced a specific, rapid, and significant increase in cytoplasmic ROS production (Figures 6B and 6C; $p < 0.05$ for type-A oligomeric samples and $p < 0.001$ for type-B oligomeric samples, respectively). The lack of ROS production from the mitochondria, which are the primary source of ROS in cells, suggests that the excessive ROS production observed in neurons and astrocytes is induced directly by the oligomeric αS species. In support of this conclusion, application of 20 μM ABSEF, a potent NADPH oxidase inhibitor, which is the main producer of ROS in both cytosol and mitochondria, was initially able to delay but not completely block the aberrant ROS production induced through uptake of the type-B oligomeric sample (Figure 6C; n = 67 cells).

Overall, therefore, these experiments show that the PK-resistant αS soluble oligomers induce much higher levels of ROS in cells than do the PK-sensitive oligomers, suggesting that the different oligomeric structures have different physiological effects. Moreover, our results are in excellent agreement with a recent study of a model protein that showed that only the addition of oligomers exposing hydrophobic surfaces to the cellular media resulted in an increase in intracellular ROS production (Zampagni et al., 2011) and in agreement with previous theoretical and experimental observations in which the degree of hydrophobic exposure in oligomeric species has been directly linked to cellular dysfunction (Bolognesi et al., 2010; Campioni et al., 2010; Cheon et al., 2007).

Conversion to Proteinase-K-Resistant Oligomers Is the Key Step in Aggregation

Of particular importance in this study has been the identification of a slow conversion from the initially formed and readily degradable oligomers to compact and highly structured oligomers that are likely to contain amyloid-like β sheet structure and have a significant fraction of the hydrophobic side chains exposed to the solvent. These latter oligomers induce much higher levels of ROS in cells than do their precursor oligomers, indicating that they are more damaging to cells as aberrant ROS production has been shown to activate apoptotic cascades that result in the death of dopaminergic neurons (Jenner and Olanow, 1998; Jenner, 2003). At the same time, increased ROS levels are thought to accelerate additional αS oligomer formation (Maries et al., 2003), reflecting the positive feedback between such processes, which can explain the cascade of events causing the development of disease. These results also suggest that the rate of the structural conversion relative to other biologically relevant processes will be a crucial factor in controlling the accumulation of damaging and degradation resistant oligomeric species. It is interesting to note in this regard that this slow structural conversion is on the same timescale as the half-life for αS turnover in vivo reported previously (Cuervo et al., 2004; Okochi et al., 2000). Together with the fact that the initially formed oligomers are highly degradable, this result indicates that there is a significant period of time for the cellular protein degradation machinery to operate and potentially time for therapeutic intervention, prior to the onset of cellular damage. Our results also indicate that stable oligomers of αS can be formed at physiologically relevant concentrations, and once even a relatively small number of β sheet oligomers are formed and free to react, the aggregation reactions can be accelerated dramatically. These findings highlight the key role that molecular chaperones and the cellular protein degradation machinery must play continually in preventing damage caused by such oligomers and hence the onset of disease (Balch et al., 2008; Morimoto, 2008).

When fibrils of αS are incubated in the absence of monomeric protein molecules, disaggregation occurs to produce the same type of oligomers as those found in the aggregation reaction, in particular damaging PK-resistant oligomers, which indicates that fibrils could act as a source of soluble species that are potentially pathogenic to neuronal cells and whose concentrations are likely to be high in the vicinity of the fibrils. This conclusion suggests that toxic agents are not generated just in the early stages of the aggregation process but also as a result of the disaggregation of amyloid fibrils, a crucial finding that needs to be taken into account when considering the origin and spread of neurodegenerative diseases and when designing therapeutic strategies, especially those based on the disaggregation of fibrils. Indeed, our results support the hypothesis that fibrils can act either to sequester misfolded potentially toxic species (Lansbury, 1999) or, depending on the conditions, to release them into the local environment, thereby unifying two apparently distinct views drawn from observations of protein aggregation in model organisms (Cohen et al., 2006).

EXPERIMENTAL PROCEDURES

Sample Preparation

The A90C mutant variant of αS was purified as a monomeric fraction from *Escherichia coli* as described previously (Hoyer et al., 2002) and labeled with either maleimide-modified AF488 or AF647 dyes (Invitrogen, Carlsbad, CA, USA) via the cysteine thiol moiety as previously reported (Thirunavukkuarasu et al., 2008). The labeled protein was purified from the excess of free dye by a P10 desalting column with Sephadex G25 matrix (GE Healthcare, Waukesha, WI, USA), concentrated using Amicon Ultra Centricons (Millipore, Billerica, MA, USA), divided into aliquots, flash frozen, and stored at −80°C. Each aliquot was thawed immediately and used only once. The degree of monomeric protein in the stock solution was assayed by smFRET as described in the Extended Experimental Procedures.

For the aggregation reactions, equimolecular concentrations of the AF488- and AF647-labeled A90C αS in Tris 25 mM (pH 7.4) and 0.1 M NaCl (with 0.01% NaN_3 to prevent bacterial growth during the experiments) were mixed to give a final volume of 300 μl, bringing the total protein concentration to 1 mg/ml (70 μM). The solutions were incubated in the dark at 37°C, with constant agitation at 200 rpm for 4–8 days, during which time aliquots were taken. At each

time point, a 2 μl aliquot was diluted 10^5-fold by serial dilution with 0.022 μm-filtered buffer (Tris 25 mM [pH 7.4] and 0.1 M NaCl) for smFRET analysis at room temperature. Glass slides were treated by incubation for 1 hr with BSA at 1 mg/ml to prevent αS aggregates from adsorbing to the surface (as shown by TIRF, see Extended Experimental Procedures), and immediately after removal of the BSA solution, 500 μl of diluted sample was placed on the slide for analysis.

For the disaggregation experiments, fibrils formed from a mixture of protein labeled with each fluorophore were washed by two cycles of centrifugation and resuspension in 500 μl of fresh buffer. Then, 300 μl of fresh monomer-free buffer solution was added to the fibrillar pellet, which was then gently resuspended and incubated at 37°C without shaking for 4 weeks. The total protein concentration in the fibril disaggregation samples was estimated to be ≤1 μM.

Single-Molecule Data Collection and Analysis

The instrumentation used for TCCD and smFRET experiments has been described in detail previously (Orte et al., 2006). For smFRET, only the 488 nm laser was used to excite the sample (Figure 1B), with a total laser power of 47.5 μW. PC-implemented multichannel scalar cards (MCS-PCS, Ortec) were used to collect photon counts from two avalanche photodiode detectors with 1 ms bin times over 8,000 channels on both MCS cards. Typically, 999 frames of 8,000 ms were collected for a total measurement time of 3 hr per aliquot. During the measurements, the microscope stage was moved at a constant scanning rate of 200 μm/sby two orthogonal DC motors (M-112.IDG, Physik Instrumente) so that the encounter rate does not depend on the size of the observed species. The photon time traces were analyzed by first setting an optimized threshold value for each channel under our conditions of measurement to remove the background noise: a 20 photon/ms bin for the donor channel and a 10 photon/ms bin for the acceptor channel. We analyzed the event data to derive the distributions of oligomer sizes and FRET values. Based on the average intensity from a blue monomer, the approximate number of monomers per oligomer event can be extracted after correcting the blue intensity of the oligomer for the fact that it is reduced by FRET and also taking into account the fact that 50% of the monomers are donors (confirmed by TCCD experiments, see Extended Experimental Procedures), using the following equation:

$$\text{Apparent oligomer size} = 2 \cdot \left(\frac{I_{DA} + I_A/\gamma}{I_{D\_monomer}} \right), \qquad \text{(Equation 1)}$$

where I_A and I_{DA} correspond to the acceptor fluorescence intensity and to the donor fluorescence intensity in the presence of acceptor, respectively; $I_{D\_monomer}$ corresponds to the average intensity of donor monomers (ca. 30 counts), and γ corresponds to a correction factor that accounts for different quantum yields and detection efficiencies of the donor and acceptor ($\gamma = 0.26$). We chose a relatively large oligomer bin size of five (i.e., 2–5 mers, 5–10 mers, etc.), both to present and analyze the data.

The FRET efficiency for each oligomer was calculated from the following expression:

$$E_{FRET} = \frac{I_A}{\gamma I_{DA} + I_A}. \qquad \text{(Equation 2)}$$

Because oligomers of a wide distribution of sizes were observed, a significant number of oligomer events occupied more than one time bin (1 ms). For these cases, the maximum brightness recorded per bin (i.e., the brightness recorded during 1 ms when the oligomer crosses the center of the confocal volume) was used to calculate both their size and FRET efficiency. Large species, that is, those corresponding to events occupying more than five bins or calculated to be more than 150 mers, were assumed to be fibrillar species and then were excluded from the analysis.

A full description of the methodology used in this study is presented in the Extended Experimental Procedures.

Live-Imaging Studies

Confocal images were obtained using a Zeiss 710 vis CLSM equipped with a META detection system and a 40× oil immersion objective. The 488nm Argon laser line was used to excite AF488-labeled αS and fluorescence emission collected between 505–550 nm (green channel) and 630–650nm (red channel). FRET measurements (excitation of AF488 and emission of AF647) were used to quantify the uptake of oligomeric and fibrillar αS because monomers are unable to undergo FRET. Illumination intensity was kept to a minimum (at 0.1%–0.2% of laser output) to avoid phototoxicity and the pinhole set to give an optical slice of ~2 μm. We recorded from a single focal plane and specifically selected regions within the cell to measure cellular uptake of labeled αS species. For confirmation, we checked that the recorded αS signal was intracellular at the end of each experiment by z-stack scanning and three-dimensional reconstruction using Zeiss software.

ROS Measurements

Fluorescence measurements were obtained on an epifluorescence inverted microscope equipped with a 20× fluorite objective. For HEt and MitoSOX measurements, a ratio of the oxidized/reduced form was measured: Excitation at 540 nm and emission recorded above 560 nm were used to quantify the oxidized form (ethidium), whereas excitation at 360 nm and emission collected from 405 to 470 was used for the reduced form (hydroethidium). All data reported in this study were obtained from at least five coverslips and 2–3 different cell and sample preparations.

For measurement of mitochondrial ROS production, cells were preincubated with MitoSOX (5 μM; Molecular Probes, Grand Island, NY, USA) for 10 min at room temperature. For measurement of cytosolic ROS production, dihydroethidium (2 μM) was present in the solution during the experiment. No preincubation ("loading") was used for dihydroethidium to limit the intracellular accumulation of oxidized products. Animal husbandry and experimental procedures were performed in full compliance with the United Kingdom Animal (Scientific Procedures) Act of 1986.

SUPPLEMENTAL INFORMATION

Supplemental Information includes Extended Experimental Procedures, six figures, and one table and can be found with this article online at doi:10.1016/j.cell.2012.03.037.

ACKNOWLEDGMENTS

We are grateful to Francisco Mateo and Priyanka Narayan for their helpful comments and discussions. N.C. is the recipient of a Human Frontier Science Program Long-term Fellowship (LT000795/2009). S.I.A.C. is supported by a Schiff Foundation Scholarship, A.O. acknowledges an ERG from the EU 7th FP, A.Y.C. was supported by a Gates Cambridge Scholarship, and M.S. was supported by a FEBS Fellowship. F.A.A. was recipient of a PhD Fellowship from the Italian Ministry of Education, University and Research, and C.W.B. is the recipient of a FP7 Marie Curie IEF grant. E.D., A.Y.A., and N.W.W. are supported by the Wellcome/MRC Parkinson's Disease Consortium grant to UCL/IoN, the University of Sheffield and the MRC Protein Phosphorylation Unit at the University of Dundee (grant number WT089698). C.M.D. and D.K. acknowledge with gratitude support from the Augustus Newman Foundation, the Wellcome Trust, the Leverhulme Trust, BBSRC (BB/E019927/1), the European Commission (project LSHM-CT-2006-037525), the Medical Research Council (UK), and the Engineering and Physical Sciences Research Council.

Received: May 16, 2011
Revised: November 1, 2011
Accepted: March 15, 2012
Published: May 24, 2012

REFERENCES

Abeliovich, A., Schmitz, Y., Fariñas, I., Choi-Lundberg, D., Ho, W.H., Castillo, P.E., Shinsky, N., Verdugo, J.M., Armanini, M., Ryan, A., et al. (2000). Mice lacking alpha-synuclein display functional deficits in the nigrostriatal dopamine system. Neuron *25*, 239–252.

Apetri, M.M., Maiti, N.C., Zagorski, M.G., Carey, P.R., and Anderson, V.E. (2006). Secondary structure of alpha-synuclein oligomers: characterization by raman and atomic force microscopy. J. Mol. Biol. *355*, 63–71.

Balch, W.E., Morimoto, R.I., Dillin, A., and Kelly, J.W. (2008). Adapting proteostasis for disease intervention. Science *319*, 916–919.

Bolognesi, B., Kumita, J.R., Barros, T.P., Esbjorner, E.K., Luheshi, L.M., Crowther, D.C., Wilson, M.R., Dobson, C.M., Favrin, G., and Yerbury, J.J. (2010). ANS binding reveals common features of cytotoxic amyloid species. ACS Chem. Biol. *5*, 735–740.

Bonini, N.M., and Giasson, B.I. (2005). Snaring the function of alpha-synuclein. Cell *123*, 359–361.

Bucciantini, M., Giannoni, E., Chiti, F., Baroni, F., Formigli, L., Zurdo, J., Taddei, N., Ramponi, G., Dobson, C.M., and Stefani, M. (2002). Inherent toxicity of aggregates implies a common mechanism for protein misfolding diseases. Nature *416*, 507–511.

Calamai, M., Canale, C., Relini, A., Stefani, M., Chiti, F., and Dobson, C.M. (2005). Reversal of protein aggregation provides evidence for multiple aggregated States. J. Mol. Biol. *346*, 603–616.

Campioni, S., Mannini, B., Zampagni, M., Pensalfini, A., Parrini, C., Evangelisti, E., Relini, A., Stefani, M., Dobson, C.M., Cecchi, C., and Chiti, F. (2010). A causative link between the structure of aberrant protein oligomers and their toxicity. Nat. Chem. Biol. *6*, 140–147.

Cheon, M., Chang, I., Mohanty, S., Luheshi, L.M., Dobson, C.M., Vendruscolo, M., and Favrin, G. (2007). Structural reorganisation and potential toxicity of oligomeric species formed during the assembly of amyloid fibrils. PLoS Comput. Biol. *3*, 1727–1738.

Chiou, A., Hägglöf, P., Orte, A., Chen, A.Y., Dunne, P.D., Belorgey, D., Karlsson-Li, S., Lomas, D.A., and Klenerman, D. (2009). Probing neuroserpin polymerization and interaction with amyloid-beta peptides using single molecule fluorescence. Biophys. J. *97*, 2306–2315.

Chiti, F., and Dobson, C.M. (2006). Protein misfolding, functional amyloid, and human disease. Annu. Rev. Biochem. *75*, 333–366.

Cohen, E., Bieschke, J., Perciavalle, R.M., Kelly, J.W., and Dillin, A. (2006). Opposing activities protect against age-onset proteotoxicity. Science *313*, 1604–1610.

Cohen, S.I., Vendruscolo, M., Welland, M.E., Dobson, C.M., Terentjev, E.M., and Knowles, T.P. (2011). Nucleated polymerization with secondary pathways. I. Time evolution of the principal moments. J. Chem. Phys. *135*, 065105.

Conway, K.A., Lee, S.J., Rochet, J.C., Ding, T.T., Williamson, R.E., and Lansbury, P.T., Jr. (2000). Acceleration of oligomerization, not fibrillization, is a shared property of both alpha-synuclein mutations linked to early-onset Parkinson's disease: implications for pathogenesis and therapy. Proc. Natl. Acad. Sci. USA *97*, 571–576.

Cuervo, A.M., Stefanis, L., Fredenburg, R., Lansbury, P.T., and Sulzer, D. (2004). Impaired degradation of mutant alpha-synuclein by chaperone-mediated autophagy. Science *305*, 1292–1295.

Ding, T.T., Lee, S.-J., Rochet, J.-C., and Lansbury, P.T., Jr. (2002). Annular alpha-synuclein protofibrils are produced when spherical protofibrils are incubated in solution or bound to brain-derived membranes. Biochemistry *41*, 10209–10217.

Dinner, A.R., Sali, A., Smith, L.J., Dobson, C.M., and Karplus, M. (2000). Understanding protein folding via free-energy surfaces from theory and experiment. Trends Biochem. Sci. *25*, 331–339.

Geddes, A.J., Parker, K.D., Atkins, E.D., and Beighton, E. (1968). "Cross-beta" conformation in proteins. J. Mol. Biol. *32*, 343–358.

Goldberg, M.S., and Lansbury, P.T., Jr. (2000). Is there a cause-and-effect relationship between alpha-synuclein fibrillization and Parkinson's disease? Nat. Cell Biol. *2*, E115–E119.

Heise, H., Hoyer, W., Becker, S., Andronesi, O.C., Riedel, D., and Baldus, M. (2005). Molecular-level secondary structure, polymorphism, and dynamics of full-length alpha-synuclein fibrils studied by solid-state NMR. Proc. Natl. Acad. Sci. USA *102*, 15871–15876.

Hoyer, W., Antony, T., Cherny, D., Heim, G., Jovin, T.M., and Subramaniam, V. (2002). Dependence of alpha-synuclein aggregate morphology on solution conditions. J. Mol. Biol. *322*, 383–393.

Hoyer, W., Cherny, D., Subramaniam, V., and Jovin, T.M. (2004). Rapid self-assembly of alpha-synuclein observed by in situ atomic force microscopy. J. Mol. Biol. *340*, 127–139.

Hong, D.P., Fink, A.L., and Uversky, V.N. (2008). Structural characteristics of alpha-synuclein oligomers stabilized by the flavonoid baicalein. J. Mol. Biol. *383*, 214–223.

Iwai, A., Masliah, E., Yoshimoto, M., Ge, N., Flanagan, L., de Silva, H.A., Kittel, A., and Saitoh, T. (1995). The precursor protein of non-A beta component of Alzheimer's disease amyloid is a presynaptic protein of the central nervous system. Neuron *14*, 467–475.

Jackson, S.E. (1998). How do small single-domain proteins fold? Fold. Des. *3*, R81–R91.

Jarrett, J.T., and Lansbury, P.T., Jr. (1992). Amyloid fibril formation requires a chemically discriminating nucleation event: studies of an amyloidogenic sequence from the bacterial protein OsmB. Biochemistry *31*, 12345–12352.

Jenner, P. (2003). Oxidative stress in Parkinson's disease. Ann. Neurol. *53* (*Suppl 3*), S26–S36, discussion, S36–S38.

Jenner, P., and Olanow, C.W. (1998). Understanding cell death in Parkinson's disease. Ann. Neurol. *44*(3, Suppl 1), S72–S84.

Junker, M., and Clark, P.L. (2010). Slow formation of aggregation-resistant beta-sheet folding intermediates. Proteins *78*, 812–824.

Kayed, R., Head, E., Thompson, J.L., McIntire, T.M., Milton, S.C., Cotman, C.W., and Glabe, C.G. (2003). Common structure of soluble amyloid oligomers implies common mechanism of pathogenesis. Science *300*, 486–489.

Knowles, T.P., Waudby, C.A., Devlin, G.L., Cohen, S.I., Aguzzi, A., Vendruscolo, M., Terentjev, E.M., Welland, M.E., and Dobson, C.M. (2009). An analytical solution to the kinetics of breakable filament assembly. Science *326*, 1533–1537.

Kocisko, D.A., Priola, S.A., Raymond, G.J., Chesebro, B., Lansbury, P.T., Jr., and Caughey, B. (1995). Species specificity in the cell-free conversion of prion protein to protease-resistant forms: a model for the scrapie species barrier. Proc. Natl. Acad. Sci. USA *92*, 3923–3927.

Kostka, M., Högen, T., Danzer, K.M., Levin, J., Habeck, M., Wirth, A., Wagner, R., Glabe, C.G., Finger, S., Heinzelmann, U., et al. (2008). Single particle characterization of iron-induced pore-forming alpha-synuclein oligomers. J. Biol. Chem. *283*, 10992–11003.

Lansbury, P.T., Jr. (1999). Evolution of amyloid: what normal protein folding may tell us about fibrillogenesis and disease. Proc. Natl. Acad. Sci. USA *96*, 3342–3344.

Lashuel, H.A., Petre, B.M., Wall, J., Simon, M., Nowak, R.J., Walz, T., and Lansbury, P.T., Jr. (2002). Alpha-synuclein, especially the Parkinson's disease-associated mutants, forms pore-like annular and tubular protofibrils. J. Mol. Biol. *322*, 1089–1102.

Luheshi, L.M., Tartaglia, G.G., Brorsson, A.C., Pawar, A.P., Watson, I.E., Chiti, F., Vendruscolo, M., Lomas, D.A., Dobson, C.M., and Crowther, D.C. (2007). Systematic in vivo analysis of the intrinsic determinants of amyloid Beta pathogenicity. PLoS Biol. *5*, e290.

MacPhee, C.E., and Dobson, C.M. (2000). Chemical dissection and reassembly of amyloid fibrils formed by a peptide fragment of transthyretin. J. Mol. Biol. *297*, 1203–1215.

Maries, E., Dass, B., Collier, T.J., Kordower, J.H., and Steece-Collier, K. (2003). The role of α-synuclein in Parkinson's disease: insights from animal models. Nat. Rev. Neurosci. *4*, 727–738.

Miake, H., Mizusawa, H., Iwatsubo, T., and Hasegawa, M. (2002). Biochemical characterization of the core structure of alpha-synuclein filaments. J. Biol. Chem. *277*, 19213–19219.

Morimoto, R.I. (2008). Proteotoxic stress and inducible chaperone networks in neurodegenerative disease and aging. Genes Dev. *22*, 1427–1438.

Nath, S., Meuvis, J., Hendrix, J., Carl, S.A., and Engelborghs, Y. (2010). Early aggregation steps in alpha-synuclein as measured by FCS and FRET: evidence for a contagious conformational change. Biophys. J. *98*, 1302–1311.

Neumann, M., Kahle, P.J., Giasson, B.I., Ozmen, L., Borroni, E., Spooren, W., Müller, V., Odoy, S., Fujiwara, H., Hasegawa, M., et al. (2002). Misfolded proteinase K-resistant hyperphosphorylated alpha-synuclein in aged transgenic mice with locomotor deterioration and in human alpha-synucleinopathies. J. Clin. Invest. *110*, 1429–1439.

Nordstedt, C., Näslund, J., Tjernberg, L.O., Karlström, A.R., Thyberg, J., and Terenius, L. (1994). The Alzheimer A beta peptide develops protease resistance in association with its polymerization into fibrils. J. Biol. Chem. *269*, 30773–30776.

Okochi, M., Walter, J., Koyama, A., Nakajo, S., Baba, M., Iwatsubo, T., Meijer, L., Kahle, P.J., and Haass, C. (2000). Constitutive phosphorylation of the Parkinson's disease associated alpha-synuclein. J. Biol. Chem. *275*, 390–397.

Onuchic, J.N., Luthey-Schulten, Z., and Wolynes, P.G. (1997). Theory of protein folding: the energy landscape perspective. Annu. Rev. Phys. Chem. *48*, 545–600.

Orte, A., Clarke, R., Balasubramanian, S., and Klenerman, D. (2006). Determination of the fraction and stoichiometry of femtomolar levels of biomolecular complexes in an excess of monomer using single-molecule, two-color coincidence detection. Anal. Chem. *78*, 7707–7715.

Orte, A., Birkett, N.R., Clarke, R.W., Devlin, G.L., Dobson, C.M., and Klenerman, D. (2008). Direct characterization of amyloidogenic oligomers by single-molecule fluorescence. Proc. Natl. Acad. Sci. USA *105*, 14424–14429.

Parihar, M.S., Parihar, A., Fujita, M., Hashimoto, M., and Ghafourifar, P. (2009). Alpha-synuclein overexpression and aggregation exacerbates impairment of mitochondrial functions by augmenting oxidative stress in human neuroblastoma cells. Int. J. Biochem. Cell Biol. *41*, 2015–2024.

Picotti, P., De Franceschi, G., Frare, E., Spolaore, B., Zambonin, M., Chiti, F., de Laureto, P.P., and Fontana, A. (2007). Amyloid fibril formation and disaggregation of fragment 1-29 of apomyoglobin: insights into the effect of pH on protein fibrillogenesis. J. Mol. Biol. *367*, 1237–1245.

Serio, T.R., Cashikar, A.G., Kowal, A.S., Sawicki, G.J., Moslehi, J.J., Serpell, L., Arnsdorf, M.F., and Lindquist, S.L. (2000). Nucleated conformational conversion and the replication of conformational information by a prion determinant. Science *289*, 1317–1321.

Spillantini, M.G., Crowther, R.A., Jakes, R., Hasegawa, M., and Goedert, M. (1998). alpha-Synuclein in filamentous inclusions of Lewy bodies from Parkinson's disease and dementia with lewy bodies. Proc. Natl. Acad. Sci. USA *95*, 6469–6473.

Tabner, B.J., Turnbull, S., El-Agnaf, O., and Allsop, D. (2001). Production of reactive oxygen species from aggregating proteins implicated in Alzheimer's disease, Parkinson's disease and other neurodegenerative diseases. Curr. Top. Med. Chem. *1*, 507–517.

Thirunavukkuarasu, S., Jares-Erijman, E.A., and Jovin, T.M. (2008). Multiparametric fluorescence detection of early stages in the amyloid protein aggregation of pyrene-labeled alpha-synuclein. J. Mol. Biol. *378*, 1064–1073.

Tokuda, T., Qureshi, M.M., Ardah, M.T., Varghese, S., Shehab, S.A., Kasai, T., Ishigami, N., Tamaoka, A., Nakagawa, M., and El-Agnaf, O.M. (2010). Detection of elevated levels of α-synuclein oligomers in CSF from patients with Parkinson disease. Neurology *75*, 1766–1772.

Vilar, M., Chou, H.T., Lührs, T., Maji, S.K., Riek-Loher, D., Verel, R., Manning, G., Stahlberg, H., and Riek, R. (2008). The fold of alpha-synuclein fibrils. Proc. Natl. Acad. Sci. USA *105*, 8637–8642.

Winner, B., Jappelli, R., Maji, S.K., Desplats, P.A., Boyer, L., Aigner, S., Hetzer, C., Loher, T., Vilar, M., Campioni, S., et al. (2011). In vivo demonstration that alpha-synuclein oligomers are toxic. Proc. Natl. Acad. Sci. USA *108*, 4194–4199.

Wood, S.J., Wypych, J., Steavenson, S., Louis, J.C., Citron, M., and Biere, A.L. (1999). alpha-synuclein fibrillogenesis is nucleation-dependent. Implications for the pathogenesis of Parkinson's disease. J. Biol. Chem. *274*, 19509–19512.

Zampagni, M., Cascella, R., Casamenti, F., Grossi, C., Evangelisti, E., Wright, D., Becatti, M., Liguri, G., Mannini, B., Campioni, S., et al. (2011). A comparison of the biochemical modifications caused by toxic and nontoxic protein oligomers in cells. J. Cell. Mol. Med. *15*, 2106–2116.

Supplemental Information

EXTENDED EXPERIMENTAL PROCEDURES

Bulk Experiments

We selected residue 90, at the C-terminal end of the central hydrophobic NAC region of αS, as an appropriate position to incorporate the fluorophores. This residue is located at the periphery of the structure thought to be highly organized in the fibrillar form (Heise et al., 2005; Vilar et al., 2008) (see Figure S1B), and consequently no major changes in the nature of the fibrillar core are expected to result from the addition of fluorophores in this position.

A preliminary characterization to confirm the covalent attachment of the Alexa fluorescence dyes to the protein was performed. The efficiency of the labeling process was checked by mass spectrometry (Mass spectrometry facility, Department of Chemistry, University of Cambridge), resulting to be higher than 95% for both Alexa Fluor (AF) dyes. No cross-linked oligomer was observed by mass spectrometry and SDS-PAGE gel (NuPAGE Novex Bis-Tris Mini Gels, Invitrogen). The conditions used for the electrophoresis were constant voltage of 200 V, and the run time was 35 min using MES SDS running buffer (Invitrogen protocol) (this conditions were always used unless otherwise indicated). To check if the presence of the dyes could distort the conformational ensemble of the monomeric protein, dynamic light scattering was used to compare the hydrodynamic properties of the wild-type (WT) and the labeled A90C-αS. Due to the laser wavelength of the instrument, only AF488 labeled protein could be measured, although similar behavior is expected for the AF647 variant. Representative size distributions of 30 μM protein in Tris 25 mM, pH 7.4, 0.1 M NaCl were recorded at 25°C on the Zetasizer Nano ZS (Malvern Instruments Ltd.) instrument at 633nm. The samples were filtered through 0.2 μm filter and the scattered light was detected at an angle of 173°. The acquired data was analyzed by Zetasizer Nano software (Malvern Instruments Ltd.). The hydrodynamic diameter found for AF488-labeled A90C-αS was identical, within the error, to that of the unlabeled WT protein (6.07 ± 0.1 and 5.89 ± 0.18 nm, respectively; see Figure S1C).

Incubations for bulk measurements were prepared exactly as for the single-molecule measurements by using 1mg/ml WT αS, AF488-labeled protein or equimolecular concentrations of AF488 and AF647-labeled protein (1 mg/ml total protein concentration) in Tris 25 mM, pH 7.4, 0.1 M NaCl, with 0.01% NaN_3 to avoid bacterial growth during sample incubation. The samples were incubated in Eppendorf tubes at 37°C under constant shaking at 200 rpm. For WT αS 10 μl aliquots at different incubation times were analyzed for thioflavin T (ThT) fluorescence, after addition of 20 μM ThT, using a Cary Eclipse fluorescence spectrophotometer (Varian). At the same time, the amount of monomeric protein was quantified by quantitative size exclusion chromatography (SEC): aliquots of 100 μl of sample were loaded onto a superdex 75 10/300 (GE Healthcare) analytical column and the absorbance followed at 280 nm. For labeled αS, the increase in ThT signal upon amyloid fibril formation could not be followed due to interferences of the fluorescence dyes in the fluorescence properties of the ThT molecule. The kinetics of fibril formation in this case was quantified by quantitative SEC and SDS-PAGE gels using the fluorescence properties of the dyes. For SDS-PAGE analysis, 2 μl of aliquots were taken and the fluorescence quantification done on a Typhoon Trio scanner (Amersham Bioscience); AF488-labeled protein was excited at 488nm and emission collected at 526nm, while AF647-labeled protein was excited at 633nm and emission collected at 670nm. The image analysis was done with ImageQuant TL v2005 software (Amersham Bioscience). For quantitative SEC analysis, 50 μl of sample at different incubation times were injected onto a superdex 75 10/300 (GE Healthcare) and the absorbance followed by 280, 488 and 647 nm.

The formation of fibrils followed by ThT or the decrease in monomeric protein followed by either SDS-PAGE gel or SEC gave very similar results, and were individually analyzed using the empirical equation generally used to fit kinetics of fibrillization (Uversky et al., 2001):

$$y = (y_i + m_i x) + \frac{(y_f + m_f x)}{1 + \exp(-((x - x_0)/\tau))} \qquad \text{Eq.S1}$$

Incubations of equimolecular concentration of AF488- and AF647-labeled αS showed a slower rate of formation of amyloid fibrils than does the unmodified protein at the same conditions (the lag-time increased from 0.5 ± 0.2 days for the unmodified protein to 1.5 ± 0.2 days for the labeled proteins) and the quantity of fibrils formed was lower than with the unmodified protein (Figures S1D and S1E). Interestingly, when only AF488-labeled protein was incubated, we found that the decrease rate of monomeric protein, as well as the final amount of monomeric protein at the end of the aggregation reaction showed intermediate values between those for the unlabeled protein and the mixture of labeled proteins (see Figure S1E), suggesting that the addition of the fluorophores in position 90 in the protein sequence could influence the stability of the fibrils, and that the larger the fluoropore, the bigger is this effect. In fact, the labeled fibrils were found to incorporate both AF488- and AF647-labeled protein, but with a slight preference for AF488 (around 70% of AF488 and 30% AF647), according to SEC analysis, measured by following the amount of monomeric protein remaining in the soluble fraction during the incubation. However, we did not observe any preference for one color at the oligomeric level according to the analysis we performed during the aggregation of the protein by single-molecule fluorescence experiments using TCCD (Two Color Coincidence Detection), indicating that this preference for monomers labeled with AF488 only takes place at the fibrillar level. The fibrils formed with the mixture of labeled proteins were also morphologically indistinguishable by TEM from those derived from the unmodified protein (Figure S1F), and showed similar proteinase K degradation profile (data not shown). In particular, there was no evidence for the formation of amorphous aggregation in either of the samples.

During the analysis of the aliquots at bulk conditions from the incubations of a mixture of AF488- and AF647-labeled protein, we observed another peak in the SEC chromatogram, apart of that of the monomeric protein, corresponding to oligomers eluting in the void volume of the column (Figure S1G). The concentration of oligomers in mass as a function of incubation time was estimated from the area of the oligomeric peak in the chromatogram obtained at different incubation times (taking the area of the monomeric peak at time 0 as a reference), and resulted to be very small at all times (being the maximum ca. 2 μM, i.e., 3% of the total protein concentration; see Figure 1D in main text). This result is in agreement with the SDS-PAGE analysis of the same aliquots, where no apparent differences were observed comparing the amount of soluble material before and after ultracentrifugation, after removing the fibrillar material.

Transmission Electron Microscopy

TEM images were obtained using a Philips CEM100 transmission electron microscope. The samples were applied on Formvar-carbon coated nickel grids and negatively stained with 2% (w/v) uranyl acetate. For the aliquots of early incubation times, the sample was applied directly onto the grids. For late incubation times, the sample was previously centrifuged for 10-15 min at 16,000 x *g*, and then the supernatant was applied to the grid and stained, while fibrillar material was applied to the grid, washed with ddH_2O, and stained.

Calculation of the Association Coefficient in smFRET Experiments

After thresholding the raw data (see Experimental Procedures in the main text), coincident events (due to oligomers exhibiting FRET) are identified and a list of all the fluorescent bursts from such events recorded during the measurement is obtained. From the number of oligomer and blue monomer events recorded per second (r_C and r_B, respectively), we obtain the association quotient, Q, for each aliquot, a measurement of the fraction of dual-labeled molecules within the sample, as previously reported (Orte et al., 2006):

$$Q = \frac{1}{2}\frac{r_C}{r_B} \qquad \text{Eq.S2}$$

The ratio of the burst rate of coincident events to the burst rate of blue monomers is divided by two to account for the rate of red monomer bursts that cannot be measured but is assumed to be the same as that of the blue monomers. In addition, the monomer burst rate is assumed to be that of the total burst rate due to the very low frequency of oligomer events compared to monomer events detected in the single-molecule experiments. The Q value was further corrected for the efficiency of detection of coincidence fluorescence; in our experiments we determined this value to be 25%, using a sample containing only dual-labeled 40bp-dsDNA (Orte et al., 2006), and for the fact that oligomeric species containing either only donor or only acceptor molecules are invisible in our approach (whose probability depends on the oligomer size according to Pascal's triangle).

Estimation of the Concentration of Oligomers at Bulk Conditions from smFRET Experiments

The concentration of oligomers (in number of species) at bulk conditions was estimated from the burst rate of oligomers detected in the smFRET experiments after correcting by the efficiency of detecting oligomers in our approach (see section above). The corrected burst rate was then transformed into concentration of oligomeric species at single-molecule conditions, using a reference to convert burst rates in concentration. As a reference, we use dual-labeled 40 base pair double-stranded DNA. The dsDNA is labeled with AF488 at the 5′-end of one strand and with AF647 at the 5′-end of the complementary strand, so the two fluorophores are far apart. For this 40bp-dsDNA model, the average molar burst rate in our setup, using the same scanning rate is 0.76 $burst.s^{-1}.pM^{-1}$. Finally, we can extrapolate the concentrations calculated at single-molecule conditions to bulk conditions by simply multiplying by the dilution factor (typically 10^5 for aggregation experiments, and 10^3 for disaggregation experiments). The stability of the oligomeric species upon dilution was checked and the effect of dilution on the number of oligomer detected was found to be very small, just 3 times higher for type A oligomers and 1.5 times higher for type B oligomers (data not shown). These values were used to correct the concentration of oligomers at bulk conditions from the smFRET analysis.

To estimate mass concentration of oligomers at bulk conditions, we used the same procedure explained above, but calculating the burst rate of protein aggregated (r_{C_mass}) instead of the burst rate in number of oligomers.

This procedure of estimating the concentration of oligomers from the burst rate in smFRET experiments using a sample of dsDNA as a reference was further proved to be valid by an alternative procedure of estimating the concentration in the single-molecule sample, based on the burst rate of aggregated protein detected and the estimation of the scanned volume in the experiment. Assuming that the probe volume is spherical (ca. 4fL, although only 25% of that volume is functional (Orte et al., 2006)), the scanned volume in the experiment can be then assumed to be cylindrical and it would depend on the scanning rate. In our experiments, the scanning rate is 200 $\mu m.s^{-1}$, which gives a scanned volume rate of 6.08×10^{-13} $L.s^{-1}$. This scanned volume rate multiplied by the burst rate of aggregated protein gives the number of oligomers per liter in the sample at single-molecule conditions. The concentration in number of oligomers per liter at bulk conditions can then be extrapolated by multiplying by the dilution factor. Both methods of calculating the concentration of oligomers at bulk conditions from smFRET measurements give rise to the same values within the experimental error.

Finally, we compared the mass concentrations of oligomeric species at bulk conditions derived from the smFRET experiments with the concentrations obtained by SEC analysis, at bulk conditions, at different incubation times during the aggregation reaction (see Figure S1G). Both the concentrations of oligomers and the kinetic curves obtained by the two methods are very similar (Figure 1D in main text), proving the validity of our smFRET approach.

Analysis of the Apparent Size- and FRET-Derived Oligomer Distribution

The apparent size and FRET efficiency for each individual oligomer can be estimated (see Experimental Procedures in the main text for a more detailed explanation). 2D histogram plots were generated, classifying each oligomer according to its apparent size and FRET efficiency value, using a bin size of 5 mers for the size histogram and a bin size of 0.05 for the FRET histogram. These values were chosen to get a statistically significant number of oligomers per bin and to account for the limit in size resolution due to the various possible combinations of AF488 and AF647 fluorophores in an oligomer and the different paths a molecule can take through the confocal volume. The data is robust to changes in the bin size used (data not shown). The oligomers were further classified in three classes according to their size: small (~2–5 mers), medium (~5–15 mers), and large (~15–150 mers) (we established 150 mers as a threshold in size for the analysis of soluble oligomers). The boundaries of the division in these three classes were chosen as to have the minimum number of classes whose FRET histograms showed the minimum number of Gaussian distributions. For each class of oligomers, the FRET efficiency histograms were globally analyzed for all incubation times recorded during the aggregation or fibril disaggregation experiment (see Figures 2C and 4B), and fitted to Gaussian distributions with constant center and width for all incubation time points. In the case of the medium size oligomers (~5–15 mers), two clear distinct FRET distributions were always observed in both aggregation and fibril disaggregation experiments, and two Gaussian distributions were needed for the fit.

From the parameters of the Gaussian distributions, the total number of oligomers at each time point and for each class of oligomers was estimated. Because the FRET efficiency histogram has a bin size of 0.05, to estimate the number of oligomers of each Gaussian distribution (which is a continuous function), each Gaussian distribution (obtained from several bins), was "transformed" into an equivalent bin of 0.05 with exactly the same area as that of the Gaussian distribution. The height of this bin corresponds to the total number of oligomers associated with the distribution. To compare the kinetics of the different oligomers with the kinetics of fibril formation obtained by SDS-PAGE gel analysis (determined then as the fraction in protein mass included in the fibrils), we need to estimate the fraction in mass of each oligomer class at bulk conditions. To do this, we first transformed the number of oligomers calculated from the analysis explained above into protein mass using an average size for each oligomer class: 4-mer for 2–5 mers, 10-mer for 5–15 mers, and 30-mer for 15–150 mer (corresponding to the approximate size at which half of the total oligomers of that class is smaller and half bigger). Then, we corrected these values for the different factors explained in the section above: the dilution factor, and the efficiency in detecting oligomers in our experiments, and the stoichiometries of the two fluorescent dyes that give FRET signal depending on the oligomer size, and the burst rate of protein aggregated for each oligomer class was calculated according to Eq.S2. This analysis was done for each oligomer class at each incubation time point, and the average values and associated standard errors for the 5 aggregation experiments performed were calculated. For the four oligomer classes (A_{small}, A_{med}, B_{med}, and B_{large}), the data was well fitted to single-exponential kinetics with a lag time:

$$y = A(1 - \exp(-(x - x_0)r)) \qquad \text{Eq.S3}$$

where A corresponds to the final concentration of oligomers reached at the end of the kinetics, r the apparent growth rate, and x_0 the lag time before start of exponential kinetics. The kinetic parameters obtained for each oligomer class are shown in Table 1 in the main text.

The possible effect of pipetting when the sample is diluted from bulk to single-molecule conditions on the number of oligomers detected was explored by comparing the number of coincident events and the oligomer distribution when the sample was pipetted using normal pipette tips and mixed by gently pipetting, with when the sample was obtained using pipette tips whose bottom part was cut (so the pipette had a bigger diameter to avoid fragmentation of large oligomers/fibrils) and the mixing was done by tube inverting. The differences in the number of coincident events and the size- and FRET-distributions were insignificant between these two methods.

Analysis of the Average Ratio of Donors and Acceptors in the Oligomeric Species by TCCD Experiments

Because we observed a slight preference of αS labeled with AF488 in the fibrils: ca. 70% of the protein incorporated in the fibrils contains AF488 and 30% AF647 according to SEC and SDS-PAGE analysis, we performed an analysis of the average ratio of number of donors and acceptor in the soluble oligomeric species detected in single-molecule experiments by Two Color Coincidence Detection (TCCD) method (Orte et al., 2006) using the same instrument as that used for the smFRET experiments.

SmTCCD measurements of aliquots of an αS aggregating sample at different incubation times were performed in the same way as for the FRET measurements, and the data of 15 measurements corresponding to different incubation times and different αS aggregation experiments were independently analyzed. Grouping all the coincident events detected in all the experiments, the average ratio of brightness in the blue and the red channel obtained was 0.906. As the labeled fibrils seem to have a ratio between blue and red labeled monomers of 2.3, we also checked if there is a size-dependence of the ratio between brightness of oligomers

detected in our experiments. For that, we calculated the size of each oligomer and we grouped all the oligomers detected in the 15 experiments by their size. The ratio between average brightness falls between 0.7 and 1.4 and without any dependence with the size of the oligomers, suggesting that the assumption of equal distribution of blue and red labeled proteins in the αS aggregates is valid at least for the soluble oligomeric species detected in our solution sm fluorescence approach. The preference for AF488-labeled molecules with respect to molecules containing AF647 is found to be then in large protein species inaccessible to our smFRET experiments, likely due to strong steric and electrostatic repulsions in fibrils composed by a high number of AF647 fluorophores, which is bigger and more negatively charged than AF488.

Limited Digestion of Protein Species with Proteinase K

The sensitivity of αS monomers, oligomers, and fibrils to proteinase K degradation was tested. To determine the sensitivity of monomers to proteinase K degradation, we used an equimolecular concentration of AF488- and AF647-labeled A90C αS of 70 μM in Tris 25 mM pH 7.4, 0.1 M NaCl; 2 μl of sample were diluted into 500 μl of buffer and 1 μl of proteinase K solution (Proteinase K from *Tritirachium album*, *Sigma-Aldrich*) was added to a final concentration of 0.01, 0.1, 0.4, 2, and 10 μg/ml (i.e., at protein:proteinase K ratios varying from 1:0.0025 to 1:2.5). The reaction sample was then incubated for 20 min at 37°C and the reactions stopped by addition of SDS-PAGE loading buffer and loaded in SDS-PAGE gels. The quantification of the level of proteinase K resistance was performed using the band in the gel corresponding to full-length monomeric αS (see Figure S3B). The gel analysis was done as explained in the previous section (bulk experiments). An αS fibrillar sample with an estimated concentration of around 30 μM was incubated with the same proteinase K concentrations in the same way as for the monomeric sample, but in this case a serine-protease inhibitors cocktail (PMSF, *Sigma-Aldrich*) was added in 1200:1 ratio with respect to the proteinase K concentration to stop the degradation reaction. For quantification of the level of degradation of each sample, we dissolved the fibrils into monomers by adding guanidine thyocianate at a 2 M final concentration and incubated the sample for 1 hr at room temperature. This concentration of guanidine thyocianate was able to dissolve the fibrils without significantly increasing the ionic strength of the samples, allowing the normal running of the protein samples in SDS-PAGE gels for quantification (see Figure S3B). High concentrations of either DMSO or urea were unable to dissolve the fibrils into monomers.

To quantify the stability of the oligomeric species, we incubated a sample of equimolar concentration AF488- and AF647-labeled A90C αS (70 μM of total concentration) in Tris 25 mM pH 7.4, 0.1 M NaCl in aggregating conditions, and after 5 days of incubation we took 2 μl aliquots, which were incubated with proteinase K exactly as was done for the monomeric and fibrillar samples. In this case, the reaction was stopped by diluting the sample 500 times in buffer, and the final sample was checked by single-molecule analysis (see Figure S3A). In all cases the final proteinase K concentration is below its active concentration, and we did not observe any degradation during the single-molecule experiment.

Time-Resolved Fluorescence Measurements of the Labeled Proteins

Time-correlated single-photon counting was performed on a PicoQuant FluoTime 200, equipped with two pulsed diode lasers of 470 nm (LDH-P-C-470) and 635 nm (LDH-P-635), combined on a fiber coupling unit and controlled by a multichannel driver (‘Sepia’ PDL-808). The repetition rate was 20 MHz, giving a FWHM around 80 ps for both laser pulses. After a polarized set at the magic angle (54.7°), and an emission monochromator, a photomultiplier was used as detector. A TimeHarp 200 PC card is used to collect data in 1,320 channels. Photon histograms were recorded until they reached typically 2×10^4 counts at the maximum at four different emission wavelengths for each dye. Decay traces were analyzed by a least-squares based deconvolution method in terms of multi-exponential functions using FluoFit software. Decay traces at the different emission wavelengths were fitted globally with the decay times linked as shared parameters, whereas the pre-exponential factors were local adjustable parameters. The quality of each fit was judged by measuring the reduced χ^2 value and the randomness in the distributions of weighted residuals and autocorrelation functions.

We measured the fluorescence decays of the labeled proteins in the soluble state and in the fibrillar state, using either 470 or 635 nm excitation to directly excite respectively the AF488 and AF647 fluorophores. The measured samples correspond to the final stage of aggregation of equimolar mixtures of AF488 and AF647-labeled synuclein. We collected fluorescence decays from both resuspended fibrils and the soluble protein (monomers and oligomers) in the supernatant of those samples.

AF647 fluorescence decay was directly probed with 635 nm excitation. AF647 typically shows bi-exponential decays usually assigned to the cis-trans equilibrium of the cyanine bridge (White et al., 2006). The average lifetime of AF647 for the soluble protein was 1.29 ± 0.01 ns, slightly higher than the reported value of 1 ns, which indicates the absence of quenching of the fluorophore upon tagging the protein. For the resuspended fibrils, another short lifetime appeared of 0.32 ± 0.01 ns that corresponded to self-quenching of the fluorophores in highly packed fibrillar material. Because this time only appears in the fibrils, we assumed negligible quenching of AF647 in the soluble oligomers.

AF488 fluorescence decay was probed with 470 nm excitation. For the soluble material, monomer and soluble oligomers, a single exponential decay was obtained, with a lifetime of 4.11 ± 0.01 ns, in good agreement with the free AF488 lifetime of 4.1 ns (Panchuk-Voloshina et al., 1999). This supports that the fluorophore is not quenched in the protein either in the monomeric or oligomeric forms. The small population of the AF488-tagged proteins forming soluble oligomers should show a decreased lifetime caused by FRET toward the acceptor AF647 dyes. However, since this population is very small it is undetectable by an ensemble-averaged technique such as time-resolved fluorimetry. This emphasizes the power of the single-molecule fluorescence techniques to reveal very rare

populations. In contrast, the AF488 dye in resuspended fibrils showed three different decay times: 4.12 ± 0.02 ns, 1.60 ± 0.10 ns, and 0.56 ± 0.02 ns. The longest lifetime corresponds to monomer and small soluble oligomers as it is very similar the lifetime from such species in the supernatant. The intermediate decay time can be attributed to bigger oligomers that show FRET from the AF488 to AF647-labeled subunits. The average FRET efficiency of this population is 0.61. The shortest lifetime corresponds to highly packed fibrils where the AF488 dyes are quenched by both FRET and self-quenching. The relative amplitudes of the three lifetimes were very variable depending on the manipulation of the sample since the resuspension and dilution of the fibrils produce breakage and fibril disaggregation.

Two-Color Total Internal Reflection Fluorescence Microscopy Measurements

Samples were prepared and diluted in the same manner as described for solution single-molecule FRET measurements, and allowed to adsorb to glass coverslips at 23°C for 5 min. The sample was illuminated through objective-type TIRF by sending the 488 nm output from a Kr/Ar laser (353-LDL-840-240, Melles Griot) through a high numerical aperture objective (60 × Plan Apo TIRF, NA 1.45, Nikon) mounted to an Nikon TE2000-U inverted microscope. A blue laser power of 1 mW was used to illuminate an area of 90 μm diameter, resulting in a good signal/noise ratio and low photobleaching. Green and red fluorescence was separated and filtered using dichroic and bandpass filters mounted within DualView optics, and imaged simultaneously on an Electron Multiplying Charge Couple Device (EMCCD; Cascade II: 512 Princeton Instruments, MA) operating at −70°C. Good image registration between the color channels was achieved as described previously (Chiou et al., 2009). Videos of 10 frames of 33 ms were acquired in 50 separate locations for each experiment using IPLab 4.0.2 software (BDBiosciences, Rockville, MD).

Data analysis was carried out using custom written routines implemented in MATLAB (The Mathworks, Natick, MA). First, an average image of each 10 frame video was obtained for each color channel and the centroid position of fluorescent objects automatically identified, as described in a previous paper (Chiou et al., 2009). In addition, the brightness of the object was calculated by summing pixels within a 7 pixel diameter circular window centered about the object.

After locating fluorescent objects, FRET species were identified using a nearest neighbor distance approach, similar to that described previously (Chiou et al., 2009). Briefly, the user specified a distance increment d, and calculated the number of red objects that were within a radius of d nm of a blue object and vice versa. This calculation was repeated for multiples of d up to a user specified maximum, D_{max}. For each object coincident at D_{max}, the red and blue brightnesses were recorded, and the FRET ratio calculated according to the same formula as for solution single-molecule experiments (Equation 3 in the main text), with the gamma-factor (γ) for this instrument calculated to be 0.273. In addition, the program recorded the brightnesses of objects which were not coincident at D_{max}. For this work it was found that true coincident objects could be identified at a distance of ~200 nm (data not shown).

To estimate the average blue monomer brightness, the brightness of blue noncoincident events from a dilute late aggregation sample, where lots of monomers were detected (in contrast to the early disaggregation sample, where the majority of the objects detected were oligomers, in agreement with solution single-molecule experiments) was examined. Spots were detected at a threshold of 5 standard deviations above background (spot detection at this threshold was in agreement with detection by eye), and a histogram formed of blue noncoincident event brightnesses (which should mean that oligomers do not contribute). The result displays a log-normal distribution with a peak maximum at 151.5 counts- taken to be the monomer brightness.

Data acquired for early fibril disaggregation (FD) and late aggregation (LA) experiments were analyzed at a threshold of 10 standard deviations above the mean. At this threshold, a large fraction of detected species showed FRET: 0.66 (FD), 0.42 (LA). The larger fraction of aggregated species detected in the early fibril disaggregation sample compared to the late aggregation sample is in agreement with solution single-molecule results. An example of the data obtained for TIRF experiments is shown in Figure S4D.

The results of FRET analysis are given in Figure S4E. The histogram of FRET efficiencies normalized to the total number of objects detected (1888(FD) and 938(LA)) showed a strong peak at a FRET efficiency of approximately 0.7 for both fibril dissolution and late aggregation experiments. The distribution extended to lower FRET efficiencies for both samples, and this was particularly pronounced for the late aggregation sample, which showed a secondary peak around 0.4-0.5, in agreement with solution single-molecule experiments. Notice that in the early disaggregation sample only one FRET distribution at high-FRET efficiencies was detected, and at the same FRET values as the high-FRET oligomers observed in solution single-molecule experiments. Using the blue brightness values for coincident events in conjunction with the calculated monomer brightness, the apparent size distribution of oligomers could be calculated (Figure S4F). This showed that larger aggregates were present in the early fibril disaggregation sample compared to late aggregation samples, as was also observed in solution smFRET experiments.

Preparation of Primary Rat Midbrain Neurons

For primary rat midbrain cultures, pups were culled by decapitation at postnatal day P2. The midbrain was removed and placed into sterile eppendorfs containing 1 ml of chilled Hanks buffered salt solution (HBSS). The HBSS was then replaced with 1 ml pre-warmed trypsin-EDTA solution and the midbrain was incubated for 5 min at 37°C. Cultures were pelleted by centrifugation at 2,000rpm for 5 min, the trypsin was gently aspirated and the cells were washed in pre-warmed HBSS, then in warm neurobasal medium containing 2% B27 supplement, 2mM glutamine, 100 I.U./ml penicillin and 100 I.U./ml streptomycin. The dissociated cells were resuspended in 1mL warm complete neurobasal medium and 3-4 drops of the cell solution were plated per well on poly-L-lysine coated coverslips in 6 well plates (6-8 coverslips per animal). The cultures were incubated in a humidified incubator at 37°C with 5% CO_2 in air for 3-4 hr,

then 2 ml pre-warmed neurobasal medium was added. Half the medium was replaced after 2 days, after which half the medium was replaced weekly. All live cell imaging experiments were performed between d12-d14 in culture.

Modeling the Data

The kinetics of the early stages of amyloid fibril formation observed in our single-molecule fluorescence experiments may be quantified in terms of a general kinetic model, shown in Figure 5A of the main text, where two classes of structures are considered explicitly. These are type B oligomers, with structural characteristics closer to amyloid fibrils, and type A oligomers, which are structurally distinct from the former species. Both types of aggregates can grow through monomer addition, and also undergo the inverse process of monomer dissociation. Furthermore, the equilibrium between monomers and aggregates is established primarily through the type A structures and no direct nucleation of type B structures from monomer occurs. The type A and type B structures of a given aggregation number can, moreover, inter-convert, with the effective nucleation of a type B species occurring via the conversion of a type A to a type B species. These considerations lead to the description of the system in terms of the master equation:

$$\frac{\partial f(t,j)}{\partial t} = 2m(t)k_+^{f,j-1}f(t,j-1) - 2m(t)k_+^{f,j}f(t,j) + 2k_{off}^{f,j+1}f(t,j+1) - 2k_{off}^{f,j}f(t,j) + k_n m(t)^{n_c}\delta_{j,n_c} - k_c^j f(t,j) + k_c^{-1,j}g(t,j) \qquad \text{Eq.S4}$$

$$\frac{\partial g(t,j)}{\partial t} = 2m(t)k_+^{g,j-1}g(t,j-1) - 2m(t)k_+^{g,j}g(t,j) + 2k_{off}^{g,j+1}g(t,j+1) - 2k_{off}^{g,j}g(t,j) + k_c^j f(t,j) - \left(k_c^{-1,j} + k_{c2}^j\right)g(t,j) \qquad \text{Eq.S5}$$

where *f(t,j)* and *g(t,j)* represent the (number) concentrations of type A and type B oligomers respectively, with polymerization number *j* at the time *t*. The free monomer concentration is given by $m(t)$. The first term in Eq.S4, $2m(t)k_+^{f,j}f(t,j-1)$ represents the increase in the number of oligomers of type A of length *j* through the growth of oligomers of length *j-1* and the second term $-2m(t)k_+^{f,j}f(t,j)$ describes the respective decrease through the growth of polymers of length *j* to a length *j+1*. The next two terms proportional to $k_{off}^{f,j}$ describe the inverse process, namely monomer dissociation, and the term $k_n m(t)^{n_c}\delta_{j,n_c}$ accounts for the creation of type A oligomers from monomers through direct primary nucleation as a reaction of the order n_c. Similar terms are present in Eq.S5 for type B species. The rate constants k_c^j, $k_c^{-1,j}$ govern the inter-conversion of type A species into type B species. Finally, the rate k_{c2}^j accounts for the possible presence of a sink from type B oligomers to larger fibrillar species that form after the bulk lag-time for amyloid fibril formation. In the most general case, the conversion rates may depend on the concentration of monomer; here we subsume these dependencies into the rate constants and note that the values obtained for the conversion rate constants are, therefore, upper bounds for those that would be observed at lower total protein concentrations. Related to this, the conversion steps may involve a change in oligomer polymerization number, but the strategy outlined below, which considers the total number of oligomers of each type, is unaffected by changes in this detail of the mechanism.

The master equations in the form of the Eqs.S4 and S5 are an infinite set of coupled non-linear differential equations and do not in general admit an exact solution but may be solved numerically.

In order to analyze our experimental data in the context of the master equation Eqs.S4 and S5, we consider the zeroeth moments of the distributions *f* and *g*, which are the observables directly measured in single-molecule fluorescence experiments: the number-concentrations $Q = \sum_j f(t,j)$, $P = \sum_j g(t,j)$. We consider the system before the bulk lag-time for fibril formation, τ_{lag}, before which the free monomer concentration has not been significantly depleted and is approximately constant at its initial value, $m(t) \approx m_{tot}$ (see Figure S1E). In this regime, many of the processes in Eqs.S4 and S5 do not have a significant effect on the number-concentrations of oligomers. In particular, in the early stages of the reaction, the reverse conversion rate can be neglected in front of the forward conversion rate, $k_c^j f(j, t<\tau_{lag}) \gg k_c^{-1,j} g(j, t<\tau_{lag})$, and the destruction of oligomers through depolymerization can be neglected in front of primary nucleation of new oligomers, $k_n m_{tot}^{n_c} \gg k_{off}^{f,n_c} f(n_c, t<\tau_{lag})$. In addition, since type B oligomers are heavily populated before the bulk lag-time, it must be that $k_c^j \gg k_{c2}^j$.

With these observations, we can obtain the principal moments in closed form under the assumption that many of the rate constants in the master equation that apply to the smaller species, populated before the lag-time and observed in our experiments, are approximately independent of the polymerization number, i.e., $k_+^j = k_+$, $k_{off}^j = k_{off}$ and $k_c^j = k_c$ for all *j* populated during the time period $t < \tau_{lag}$. The resulting rate constants represent averaged values over the oligomer size distributions, and allow representative timescales to be obtained from experimental data for each process. These simplifications allow the sums for the polymer number concentrations to be written out in closed form, with the terms in the elongation and depolymerization rates forming telescopic sums that vanish, leaving the (ordinary) differential equation system:

$$\frac{dQ}{dt} = k_n m_{tot}^{n_c} - k_c Q \qquad \text{Eq.S6}$$

$$\frac{dP}{dt} = k_c Q \qquad \text{Eq.S7}$$

These equations may be solved analytically to yield:

$$Q=\frac{k_n}{k_c}m_{tot}^{n_c}\left(1-e^{-k_c t}\right) \quad \text{Eq.S8}$$

$$P=\frac{k_n}{k_c}m_{tot}^{n_c}\left(k_c t+e^{-k_c t}-1\right) \quad \text{Eq.S9}$$

where the initial conditions corresponding to a purely monomeric system, $Q(0) = P(0) = 0$, have been used. The leading order dependencies for $t \ll 1/k_c$ for the number-concentrations Q and P can be obtained as:

$$Q\approx k_n m_{tot}^{n_c} t+\mathrm{O}\left(t^2\right) \quad \text{Eq.S10}$$

$$P\approx\frac{1}{2}k_c k_n m_{tot}^{n_c} t^2+\mathrm{O}\left(t^3\right) \quad \text{Eq.S11}$$

It is also interesting to note that in the absence of monomer depletion, Q tends to the limit $k_n m_{tot}^{n_c}/k_c$ for $t \to \infty$.

Prior to significant depletion of the free monomer, the primary nucleation reaction order, n_c, does not influence the observables and we may define an effective nucleation rate with the same dimensions as the conversion rate, $k_n' = k_n m_{tot}^{n_c - 1}$. Interestingly, the results Eqs.S10 and S11 show that the effect of the kinetic parameters k_n' and k_c can be robustly decoupled at early times. The initial gradient of Q is determined predominantly by k_n', and once this is fitted it allows k_c to be determined from the early time form of P.

The results derived in Eqs. S4–S11 allow the constants k_n' and k_c to be determined from the number concentrations of type A and type B oligomers prior to the bulk lag-time for fibril formation. Using the analytical results Eqs.S8 and S9 we are able to fit the single-molecule fluorescence experimental data up to the lag-time and obtain good fits using the two kinetic parameters k_n' and k_c. The fit, shown in Figure 5B of the main text, determines the microscopic kinetic rate constants as $k_n' = 4\cdot 10^{-8} s^{-1}$ and $k_c = 5\cdot 10^{-6} s^{-1}$. We note that for early times the rate of primary nucleation is faster than the rate of conversion of type A to type B oligomers, $k_n' m_{tot} \gg k_c Q$.

In order to understand the time course of the reaction beyond the bulk lag-time for fibril formation, we observe that the terms from the master equation Eqs.S4 and S5 that are not significant at early times can become important. In particular, the reverse conversion of type B to type A oligomers, the destruction of oligomers by depolymerization, and the formation of large fibrillar species observed in bulk experiments become significant at later times.

SUPPLEMENTAL REFERENCES

Chiou, A., Hägglöf, P., Orte, A., Chen, A.Y., Dunne, P.D., Belorgey, D., Karlsson-Li, S., Lomas, D.A., and Klenerman, D. (2009). Probing neuroserpin polymerization and interaction with amyloid-beta peptides using single molecule fluorescence. Biophys. J. *97*, 2306–2315.

Heise, H., Hoyer, W., Becker, S., Andronesi, O.C., Riedel, D., and Baldus, M. (2005). Molecular-level secondary structure, polymorphism, and dynamics of full-length alpha-synuclein fibrils studied by solid-state NMR. Proc. Natl. Acad. Sci. USA *102*, 15871–15876.

Orte, A., Clarke, R., Balasubramanian, S., and Klenerman, D. (2006). Determination of the fraction and stoichiometry of femtomolar levels of biomolecular complexes in an excess of monomer using single-molecule, two-color coincidence detection. Anal. Chem. *78*, 7707–7715.

Panchuk-Voloshina, N., Haugland, R.P., Bishop-Stewart, J., Bhalgat, M.K., Millard, P.J., Mao, F., Leung, W.Y., and Haugland, R.P. (1999). Alexa dyes, a series of new fluorescent dyes that yield exceptionally bright, photostable conjugates. J. Histochem. Cytochem. *47*, 1179–1188.

Uversky, V.N., Li, J., and Fink, A.L. (2001). Evidence for a partially folded intermediate in alpha-synuclein fibril formation. J. Biol. Chem. *276*, 10737–10744.

Vilar, M., Chou, H.T., Lührs, T., Maji, S.K., Riek-Loher, D., Verel, R., Manning, G., Stahlberg, H., and Riek, R. (2008). The fold of alpha-synuclein fibrils. Proc. Natl. Acad. Sci. USA *105*, 8637–8642.

White, S.S., Li, H., Marsh, R.J., Piper, J.D., Leonczek, N.D., Nicolaou, N., Bain, A.J., Ying, L., and Klenerman, D. (2006). Characterization of a single molecule DNA switch in free solution. J. Am. Chem. Soc. *128*, 11423–11432.

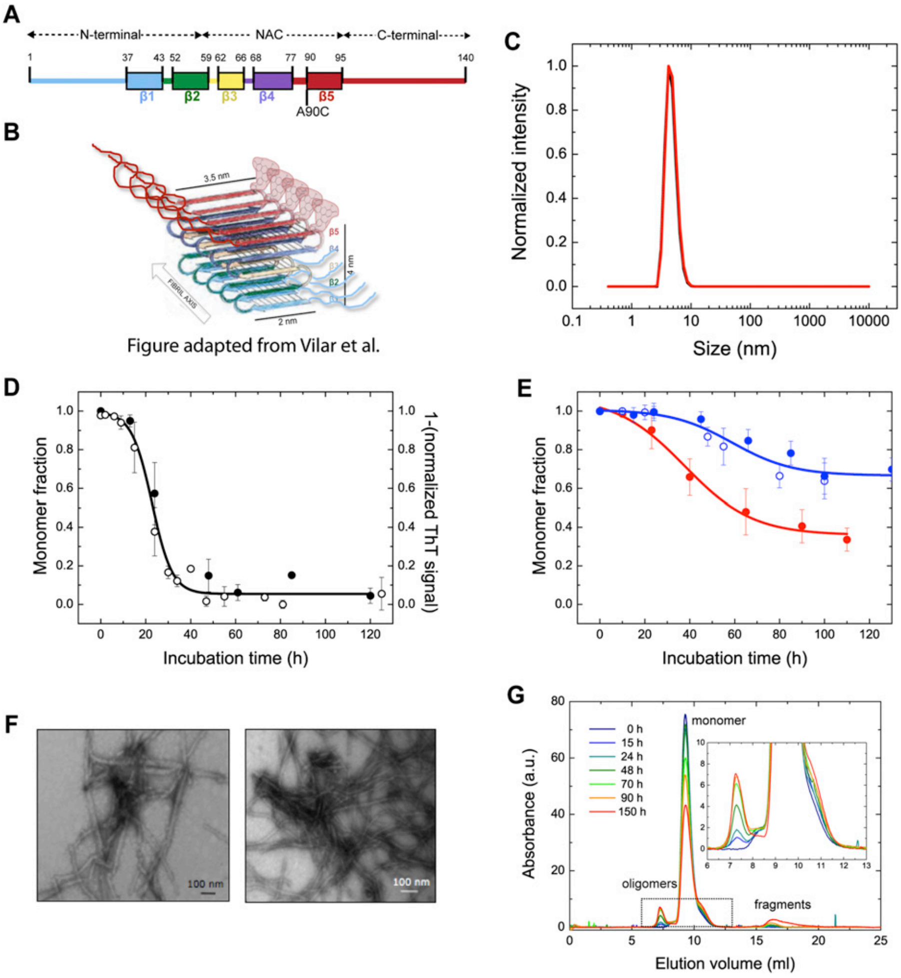

Figure S1. Characterization of Labeled A90C αS at Bulk Conditions, Related to Figure 1

(A) αS aminoacidic sequence, highlighting the three main regions: N-terminal (residues 1-60), NAC (61-95) and C-terminal (95-140); and the five regions proposed to form the strands of the beta-sheet sandwich core of the fibrillar structure (Vilar et al., 2008): strand β1 comprising residues 37-43; strand β2, 52-59; strand β3, 62-66; strand β4, 68-77 and strand β5, 90-95. The fluorescence dyes were covalently attached to position 90 in the sequence by cysteine chemistry.

(B) The fold of αS fibrillar structure proposed by Vilar et al. (Vilar et al., 2008) is shown. The fluorescence dyes would be positioned parallel at the periphery of the fibril core according to this model.

(C) Dynamic light scattering derived size distribution of unincubated AF488 A90C αS (red line) compared with unlabeled WT protein (black line). Representative size distribution of 30 μM protein concentration in Tris 25 mM, pH 7.4, 0.1 M NaCl at 25°C measured on the Zetasizer Nano ZS instrument at 633nm. The distribution represents the average of 4 measurements and the intensity was normalized and weighted by the particle size. The influence of AF647 could not be measured with our DLS instrument due to the direct absorption of the laser light (633 nm) by the fluorophore, although very similar results are expected.

(D) The kinetics of fibril formation for the unlabeled protein was independently analyzed by the addition of Thioflavin T (ThT) to the reaction sample at different incubation times (open circles: the signal was normalized and 1-(normalized signal) was plotted to compared with the kinetics of the depletion of the monomeric protein analyzed by SEC). The decrease in monomeric protein during incubation was also estimated by quantitative SEC analysis after centrifuging the samples to remove the insoluble material (closed circles) or after ultracentrifugation to remove big soluble oligomers, but no differences were observed. The lag time and apparent kinetic rate were obtained. Error bars represent SEM.

(E) For the case of labeled protein, analysis using ThT cannot be applied, since the fluorescence increase of ThT molecules upon binding to the amyloid fibrils is significantly reduced, probably due to fluorescence quenching and/or FRET between ThT and the Alexa fluorophores. For this reason, the kinetics of fibril formation was followed by quantitative SEC (closed circles; see Figure S1G) and SDS-PAGE gel (open circles), where the soluble protein material was quantified by the spectroscopic properties of the Alexa fluorophores. The aggregation of mixed AF488 and AF647-labeled protein is shown as blue circles, and the aggregation corresponding to AF488-labeled protein incubated alone is shown in red. Error bars represent SEM.

(F) TEM images show that the morphology of the labeled fibrils formed (image on the right) is very similar to those formed with unmodified protein (image on the left). Amorphous aggregates were not observed in any case.

(G) Oligomers were detected and analyzed by quantitative SEC as a function of incubation time. Three peaks were observed in the chromatogram: a peak eluting at 7 ml, which corresponds to the column void volume, and then to oligomeric species, a large peak eluting at 9.3 ml which corresponds to the monomeric protein, and a third peak at eluting volumes bigger than 15 ml, corresponding to some fragments of the protein generated upon incubation. The concentration of both monomeric and oligomeric fractions of the protein at the different incubation times recorded were estimated from the area of the peaks, taking into account the known initial concentration of protein and that at time zero, all the protein remains monomeric.

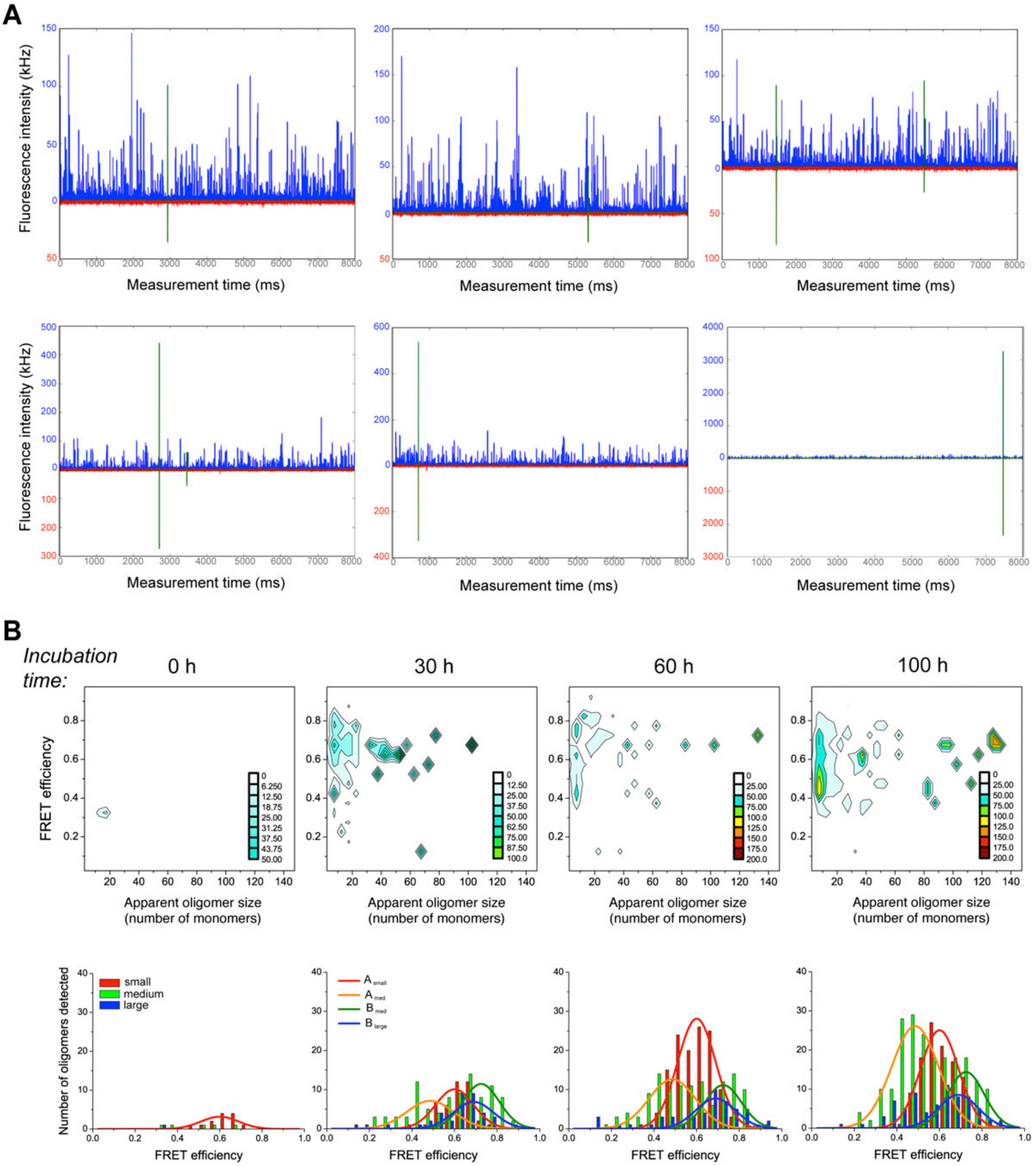

Figure S2. Aggregation Kinetics of αS, Related to Figure 2

(A) Some examples of the raw data obtained in smFRET experiments: the fluorescence intensity recorded for AF488-labeled molecules is in blue, the fluorescence intensity recorded for AF647-labeled molecules in red, and the coincident events in both channels are highlighted in green. In smFRET experiments, AF488-labeled molecules are directly excited by a 488nm-laser so both monomeric and oligomeric species containing this fluorophore are detected in the blue channel (non-coincident and coincident event, respectively). However, the AF647-labeled molecules in the oligomeric species are indirectly excited by FRET from excited AF488-labeled molecules, and therefore only oligomeric species are detected in the red channel.

(B) Aggregation of αS at physiologically relevant concentrations. The same type of experiment and analysis was carried out at 6 μM protein concentration, a value close to the proposed physiological concentration of the protein in cells, but below the critical concentration for fibrillization found in vitro. With this concentration, after incubating the protein sample for more than 150 hr, no fibrils were detectable by TEM nor reduction in the amount of soluble protein according to SDS-PAGE gel analysis, although oligomeric species were detected by smFRET experiments. In the top panels, 2D plots corresponding to the mass distribution of oligomers at different incubation times as a function of apparent oligomer size (*x* axis) and FRET efficiency (*y* axis) are represented. In the bottom panels, FRET-derived distributions of the three size classes of oligomers: small (red bars), medium (green bars) and large (blue bars) are plotted. The FRET distributions at different incubation times were globally fitted to Gaussian functions (continuous lines) to estimate the mass fraction of each class of oligomers as a function of incubation time.

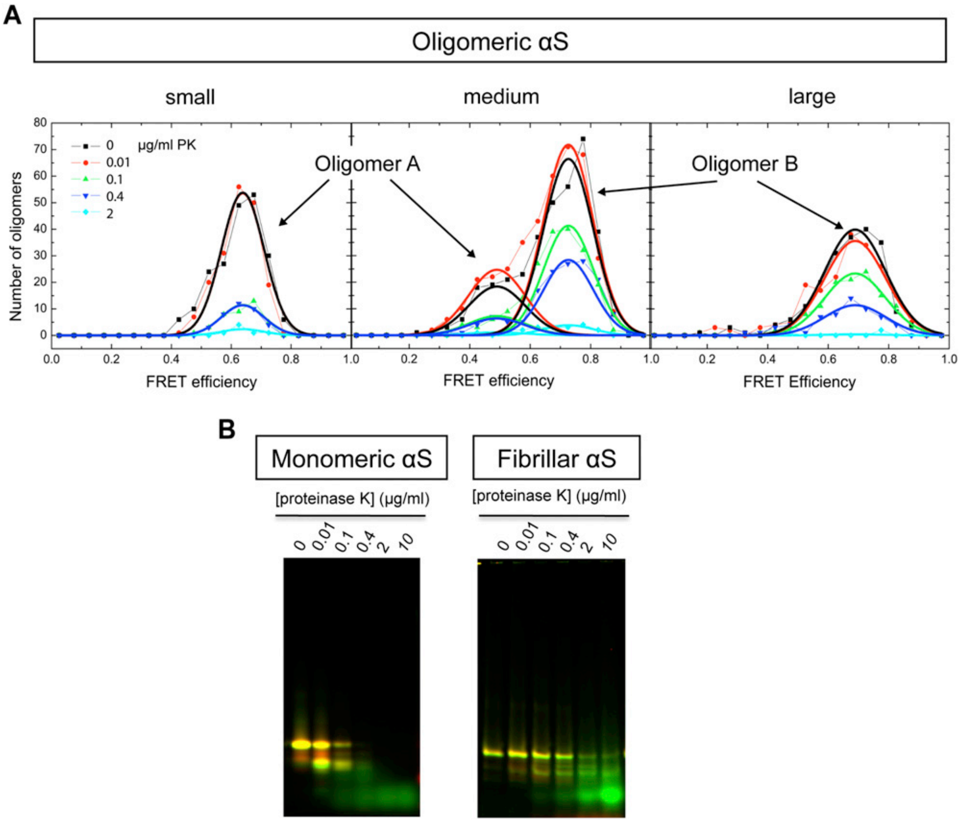

Figure S3. Analysis of the Stability of the Different Protein Species against Proteinase K Degradation, Related to Figure 3

(A) Incubations of an aliquot of an aggregation sample at late incubation times (around 100 h) with different concentrations of proteinase K were analyzed by single-molecule fluorescence to determine the stability of the different oligomeric species against proteinase K degradation. The pair of A_{small} and A_{med} distributions shows similar dependence on proteinase K concentration, as well as for the pair of B_{med} and B_{large} distributions, although this latter pair has much higher resistance to proteinase K.

(B) The stability to proteinase K degradation of monomeric and fibrillar forms of αS was estimated from the disappearance of the band corresponding to full-length monomeric in a SDS-PAGE gel. Different concentrations of proteinase K were used as indicated in the figure. All samples from monomeric, fibrillar and oligomeric forms of αS were treated exactly in the same way for direct comparison.

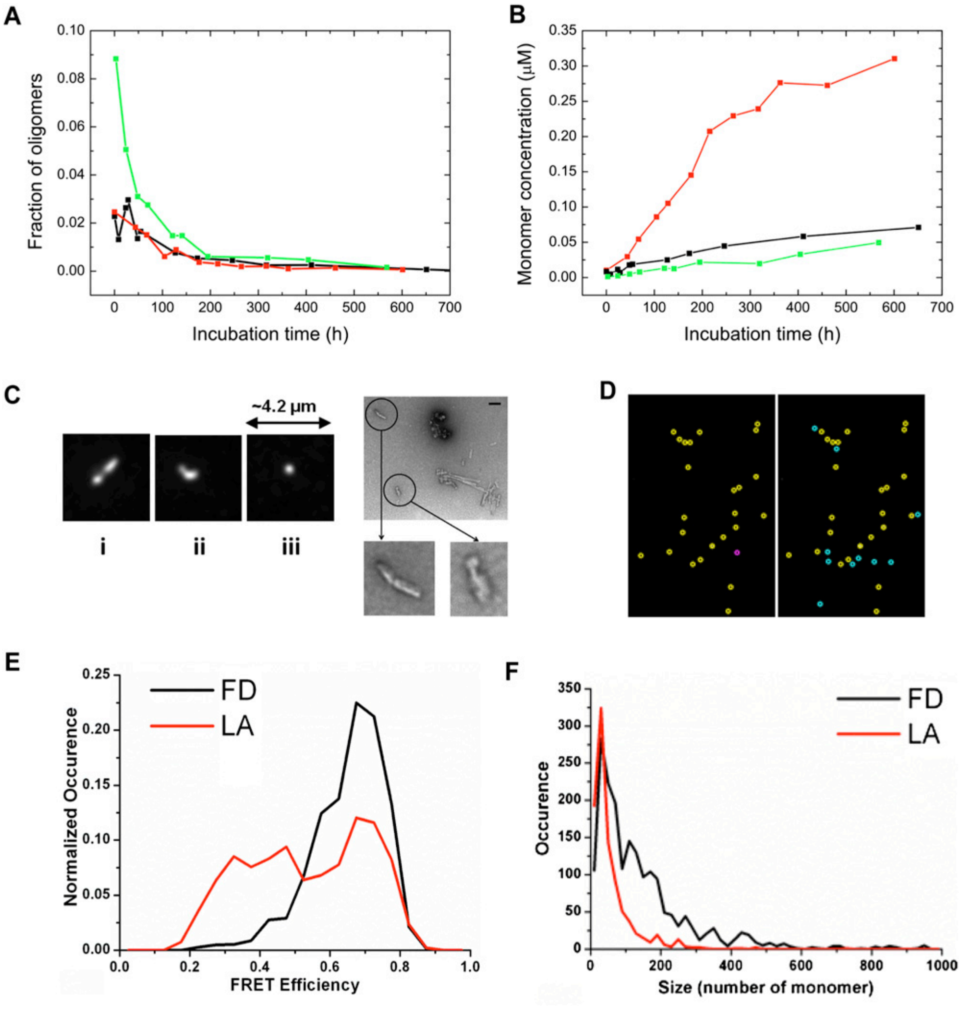

Figure S4. Fibril Disaggregation Experiments, Related to Figure 4

Both the kinetics of the fraction of oligomers (A) and the monomer concentration of the solution (B) as a function of the incubation time are qualitatively well reproduced between smFRET experiments of fibril disaggregation reactions (the three sets of data correspond to three different fibril disaggregation experiments carried out in the same conditions).

(C) Single- molecule Dual-Color Total Internal Reflectance Fluorescence (TIRF) images of αS oligomers obtained from a fibril disaggregation experiments (the three images on the left). The oligomers often displayed uneven fluorescence along their length (i) and bent/branched structures (ii). Others appeared punctate, either indicating a size smaller than the diffraction limit or oligomers standing upright in the TIRF field. The size and shape of large oligomers observed by TIRF are very similar with those seen by TEM (images on the right). Scale bar = 100 nm. Zoomed images showed branched/bent structure and aggregates with uneven width along their length, which could correspond to fluorescent structures shown in the panels on the left.

(D) Example of processed data for fibril dissolution experiment with the red channel data on the left half of the image and blue channel data on the right. Non-coincident red and blue events are marked with pink and blue circles respectively. Events, which are coincident within 500 nm, are marked with yellow circles.

(E) Histogram of FRET efficiencies for early fibril dissolution (FD) and late aggregation samples (LA). Values are normalized to the total number of detected objects: FD = 1888, LA = 938. F) Apparent oligomer size distribution showing greater size of oligomers for fibril dissolution (FD) sample compared to late aggregation (LA) sample.

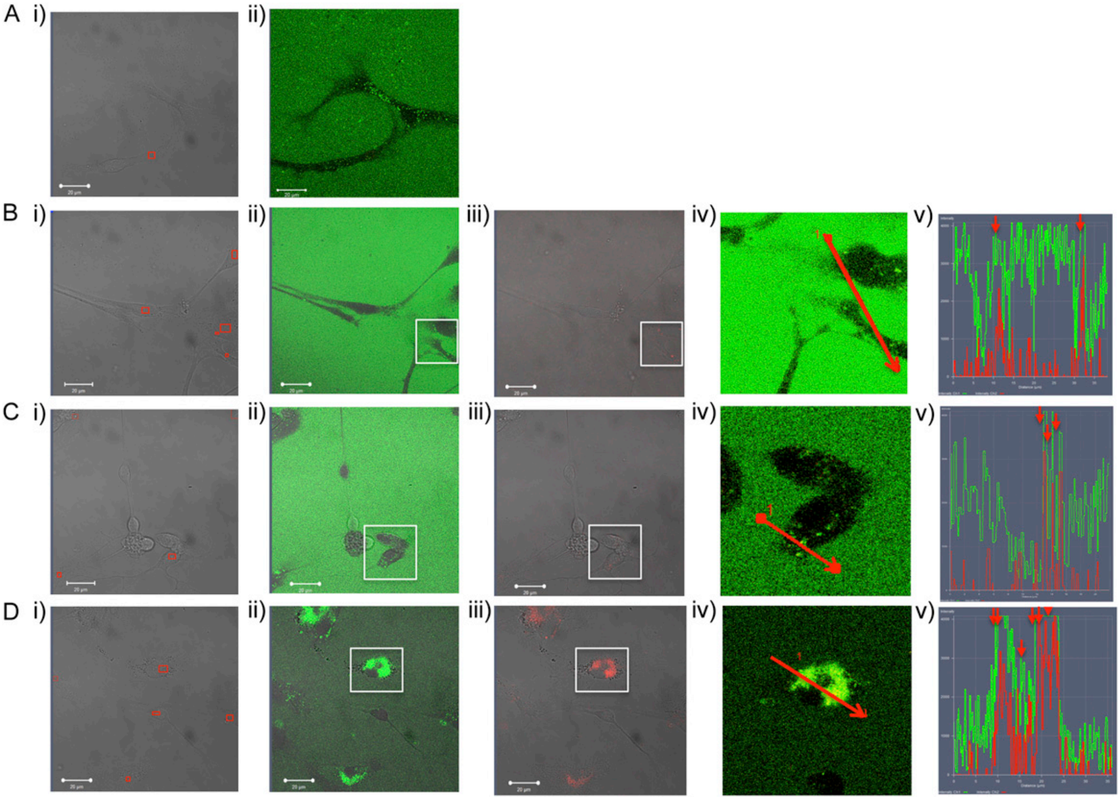

Figure S5. Cell Uptake of αS Species, Related to Figure 6

(A) Monomeric sample, (B) oligomeric A sample, (C) oligomeric B sample, and (D) fibrillar sample.

(i) Phase contrast images of rat primary neurons for each protein sample. Boxed areas show the cytoplasmic regions of cells utilized to record αS uptake. Cytoplasmic regions were specifically selected to ensure species uptake and not plasma membrane absorption was being measured.

(ii) Fluorescence signal of AF488, exciting at 488nm after the addition of each αS species sample.

(iii) Fluorescence signal of AF647, exciting the sample at 488nm to identify uptake of oligomeric and fibrillar species by FRET (monomers are unable to undergo FRET).

(iv) Magnified merged image of boxed area in corresponding images ii and iii.

(v) Profile of AF488 and AF647 fluorescence emission intensities along the red arrow in image iv. Simultaneous increase of both AF488 and AF648 signals, indicated by arrows, identify FRET signal within cell body. Images iii – v are absent for monomeric αS sample (A) because the monomeric species do not FRET.
